# Morphological and histochemical identification of telocytes in adult yak epididymis

**DOI:** 10.1038/s41598-023-32220-4

**Published:** 2023-03-31

**Authors:** Dapeng Yang, Ligang Yuan, Shaoyu Chen, Yong Zhang, Xiaojie Ma, Yindi Xing, Juanjuan Song

**Affiliations:** 1 College of Veterinary Medicine, Gansu Agricultural University, Lanzhou, 730070 China; 2 Key Laboratory of Animal Reproductive Physiology and Reproductive Regulation of Gansu Province, Lanzhou, 730070 China

**Keywords:** Biochemistry, Cell biology, Zoology

## Abstract

Telocytes (TCs) are a newly discovered type of mesenchymal cell that are closely related to the tissue’s internal environment. The study aimed to investigate the morphological identification of TCs in the epididymis of adult yak and their role in the local microenvironment. In this study, transmission electron microscopy (TEM), scanning electron microscopy, immunofluorescence, qRT-PCR, and western blotting were used to analyze the cell morphology of TCs. The results showed that there are two types of TCs in the epididymal stroma of yak by TEM; one type is distributed around the capillaries with full cell bodies, longer TPs, and a large number of secretory vesicles; the other is distributed outside the basement membrane with irregularly long, striped, large nuclei and short telopodes (TPs). In addition, these TCs formed complex TC cell networks through TPs with epididymal interstitial capillaries and basal fibroblasts. TCs often appear near the capillaries and basement membrane by special staining. The surface markers of TCs (CD34, vimentin, and CD117) were positively expressed in the epididymal stroma and epithelium by immunohistochemistry, and immunofluorescence co-expression of vimentin + CD34 and CD117 + CD34 was observed on the surface of TCs. The trends in the mRNA and protein expression of TCs surface markers revealed expression was highest in the caput epididymis. In summary, this is first report of TCs in the epididymis of yak, and two phenotypes of TCs were observed. The existence and distribution characteristics of TCs in the epididymis of plateau yaks provide important clues for further study of the adaptation to reproductive function in the plateau.

## Introduction

Telocytes (TCs) are a newly discovered type of mesenchymal cells with unique morphological characteristics that have a long cytoplasmic extensions called telopodes (TPs). TPs are slender, long, and varied in number. Their shapes are mainly irregular ellipsoid, pear, and spindle; they are rich in mitochondria, the endoplasmic reticulum, and have a secretory function^1–3^. The unique morphological characteristics of TCs make them different from other mesenchymal cells. The TPs are in close contact with blood vessels, nerve bundles, and local immune system cells through organ matrix distribution, forming a network between tissues; this network was considered to be the structural basis for cell communication^4,5^. Furthermore, TCs may also establish unique spatial relationships with a variety of cells, including adjacent parenchymal cells and other cells in the interstitial compartment, and are considered to regulate the dynamic balance of the local microenvironment by contacting or releasing secretory vesicles between cells^6–8^. These secretory vesicles are considered to be “messengers” of substance exchange and signal transduction between TCs and other cells^9,10^, and are also an important reason why TCs are thought to be secretory cells. As early as a century ago, a special cell group in the muscular layer of the human intestinal tract was discovered by Cajal and named as “interstitial neurons”^11^. Telocytes were named by Popescu and Faussone-Pellegrini at the beginning of this century^1^. For many years, research on telocytes was based on imaging their ultrastructure under a transmission electron microscope, so transmission electron microscopy is considered the “gold standard” for research into TC morphology.

TCs have been found in the human myocardium and gallbladder; CD34, c-kit (CD117), and vimentin were co-expression in TCs, and involved in stem cell differentiation, the coordination of new angiogenesis, and the regulation of paracrine function in the interstitial tissue^12,13^. Furthermore, TCs were abundant in the mouse lung and rat kidney^14,15^. TCs were found in the fish brain^16^ and in poultry skin^17^. TCs have been discovered to be involved in many functional aspects in recent years, including cell regeneration^18^, inhibition of apoptosis^19^, inflammation repair^20^, cell communication^21^, angiogenesis^22,23^, and stem cell function^19^. In previous studies, CD34, CD117, and vimentin were widely considered effective markers of TCs^24–26^. The proteins are also widely used to locate and screen TCs. CD34 is a marker receptor found on the surface of mesenchymal stem cells; it is also a highly glycosylated type I transmembrane glycoprotein expressed on the surface of hematopoietic stem/progenitor cells of humans and other mammals^27–29^.

CD117 (C-kit) is a marker of stem cells/progenitor cells in the heart^30^ and is considered one of the effective markers of TCs^31^. Vimentin, a conserved type III intermediate filament protein, is often found in fibroblasts, vascular endothelial cells, neutrophils, and macrophages, and is abundantly expressed in glomeruli, tubules, and renal interstitial cells^32^. Vimentin is a marker of mesenchymal phenotype and an important cytoskeletal protein. It is generally expressed only in mesenchymal cells and is closely related to the growth, invasion, and metastasis of tumor cells^33^. For a long time, studies of TCs have been based on some stem cell characteristics, with the stem cell surface markers such as CD34 and CD117 generally considered the marker proteins of TCs. Vimentin is a phenotypic marker of interstitial tissue, which is often used to locate TCs in the interstitial tissue.

Yak has a reputation as a ‘plateau boat’ and ‘omnipotent livestock’ in the Plateau pastoral area^34,35^. The internal environment and functions of epididymis affected by high altitude hypoxia environment, while the epididymis is an important place for sperm maturation, processing, and storage^36,37^. For example, the activity of the enzyme acrosome and its change in reactivity in hypoxic conditions damages sperm fertilization ability^38–40^. Therefore, our study aimed to determine the distribution location of TCs in the epididymis of yak and analyzed the ultrastructure of TCs, to provide new clues or information as to the function of TCs in plateau animals.

## Results

### Ultrastructural characteristics of yak epididymis TCs under TEM

**Figure 1:**
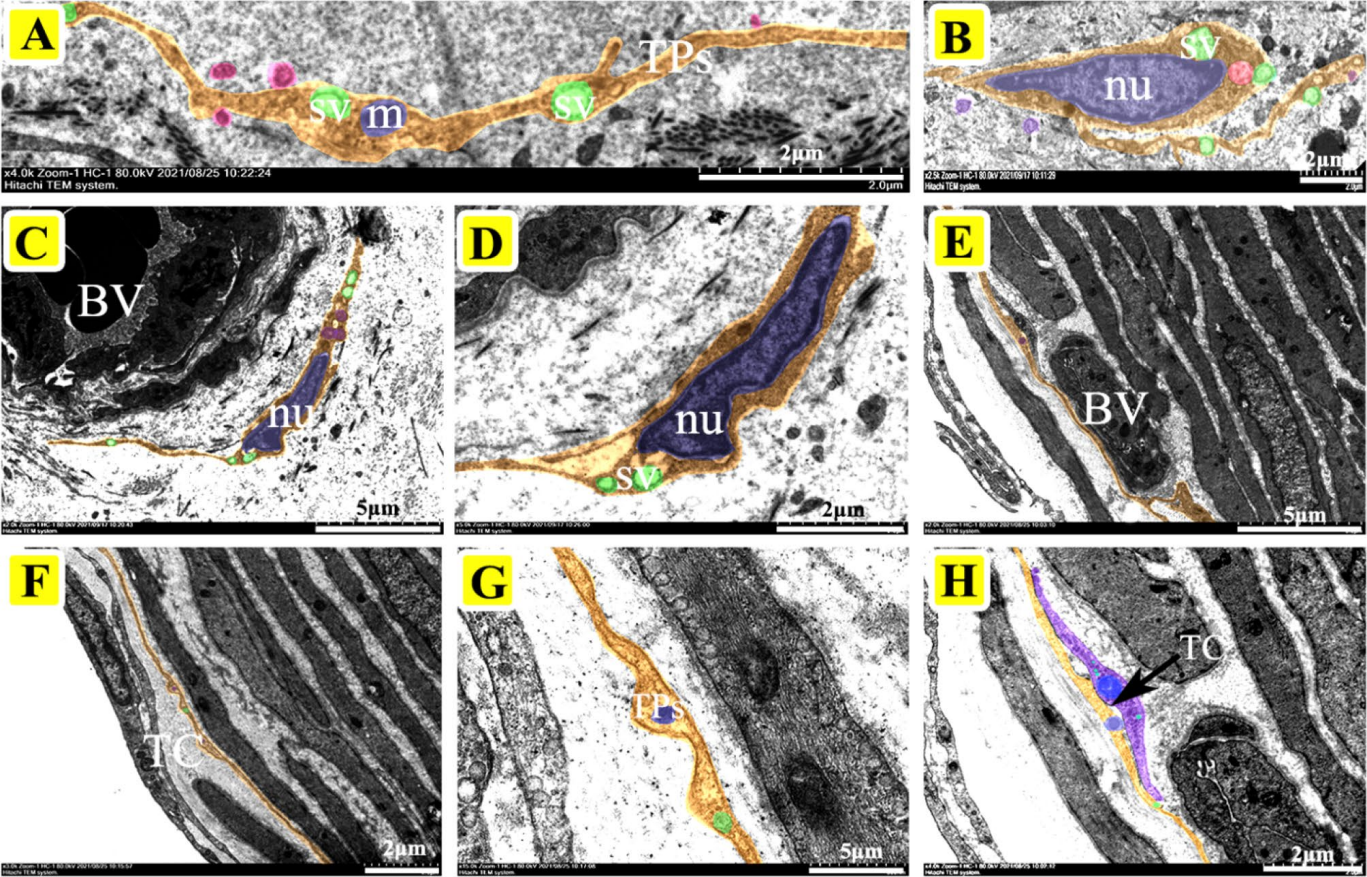
The localization of telocytes in the caput epididymis of yak by TEM. (**A**) Ultrastructure of TPs. The TPs have a long strip- or bead-like structure, which is characterized by the alternating appearance of thick protrusions and slender podomers. There are abundant secretory vesicles and mitochondria in TPs (Scale bar = 2 µm). (**B**) The TCs consist of a large spindle nuclei and a number of secretory vesicles, mitochondria, and lysosomes (Scale bar = 2 µm). (**C**, **D**) The outline of TCs was clearly visible and distributed around the interstitial capillaries of yak caput, which was composed of spindle-shaped nuclei and long TPs (**C**: Scale bar = 5 µm, **D**: Scale bar = 2 µm). (**E**, **F**) The TCs located outside the peritubular connective tissue and the TPs connected to the capillary wall to form a special network (**E**: Scale bar = 5 µm, **F**: Scale bar = 5 µm). (**G**, **H**) The TCs distributed in the intertubular stroma, had slender and coiled TPs that contacted with peritubular myoid cells and fibroblasts (**G**: Scale bar = 5 µm, **H**: Scale bar = 2 µm). BV: blood vessel, nu: nucleus, SV: secretory vesicles, m: mitochondria.

**Figure 2:**
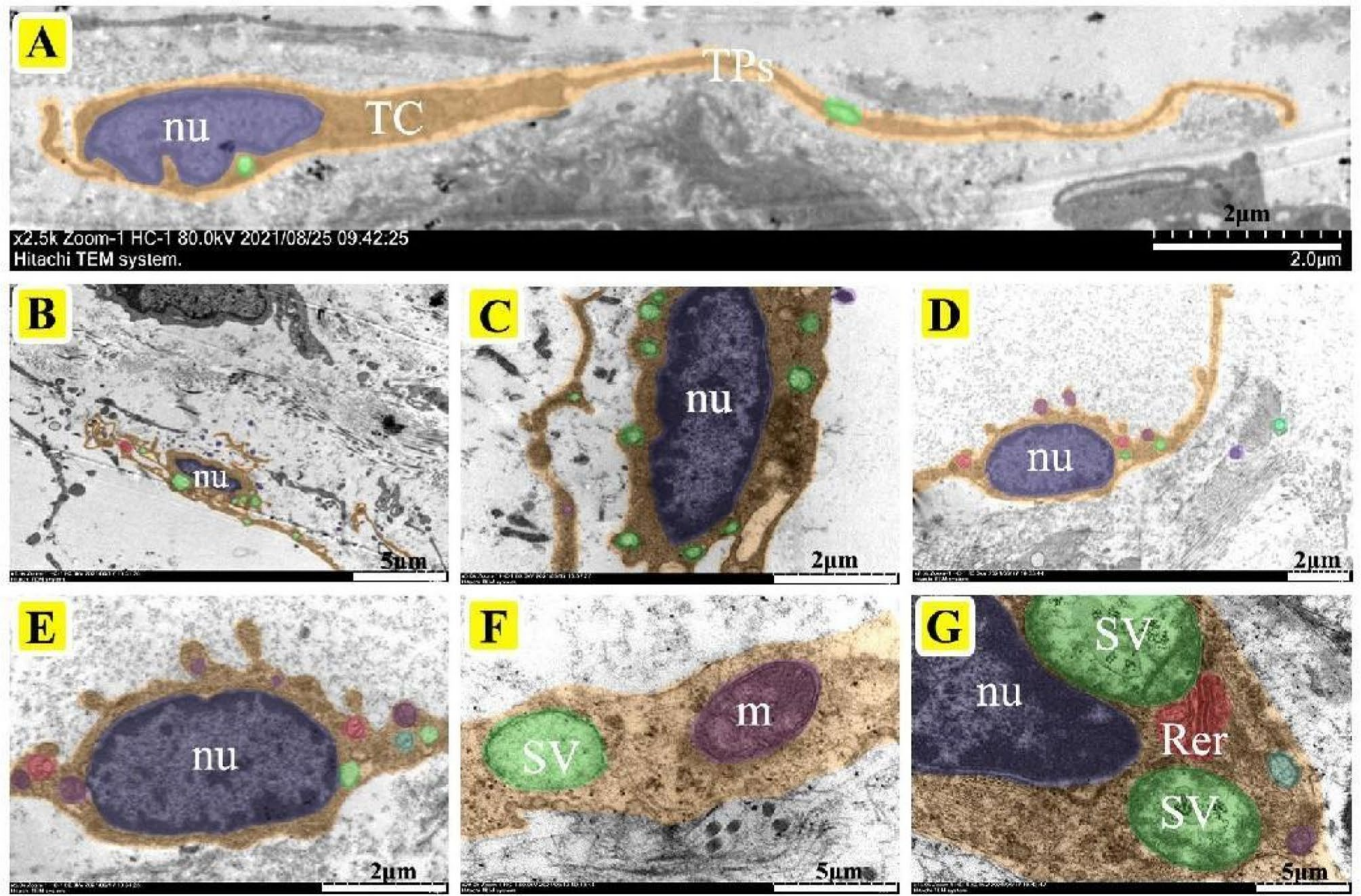
The localization of telocytes in the corpus epididymis of yak by TEM. (**A**) The slender TPs were similar to the TCs of the caput epididymis, which alternated between thick processes and slender podomers. Secretory vesicles and mitochondria were distributed around the TPs (Scale bar = 5 µm). (**B**, **C**) Telocytes are distributed in the interstitium of the corpus, with a large body and slender TPs (Scale bar = 2 µm). (**D**, **E**) The TCs are clearly visible in the interstitium of the corpus with ellipsoidal nuclei and slender TPs. A large number of secretory vesicles were distributed throughout the cells (Scale bar = 5 µm). (**F**) The secretory vesicles and mitochondria in TPs under high magnification. (**G**) The nucleoli of TCs were clearly visible under high magnification and were rich in extranuclear secretory vesicles and rough endoplasmic reticulum (Scale bar = 5 µm). nu: nucleus, SV: secretory vesicles, m: mitochondria, Rer: rough endoplasmic reticulum.

**Figure 3:**
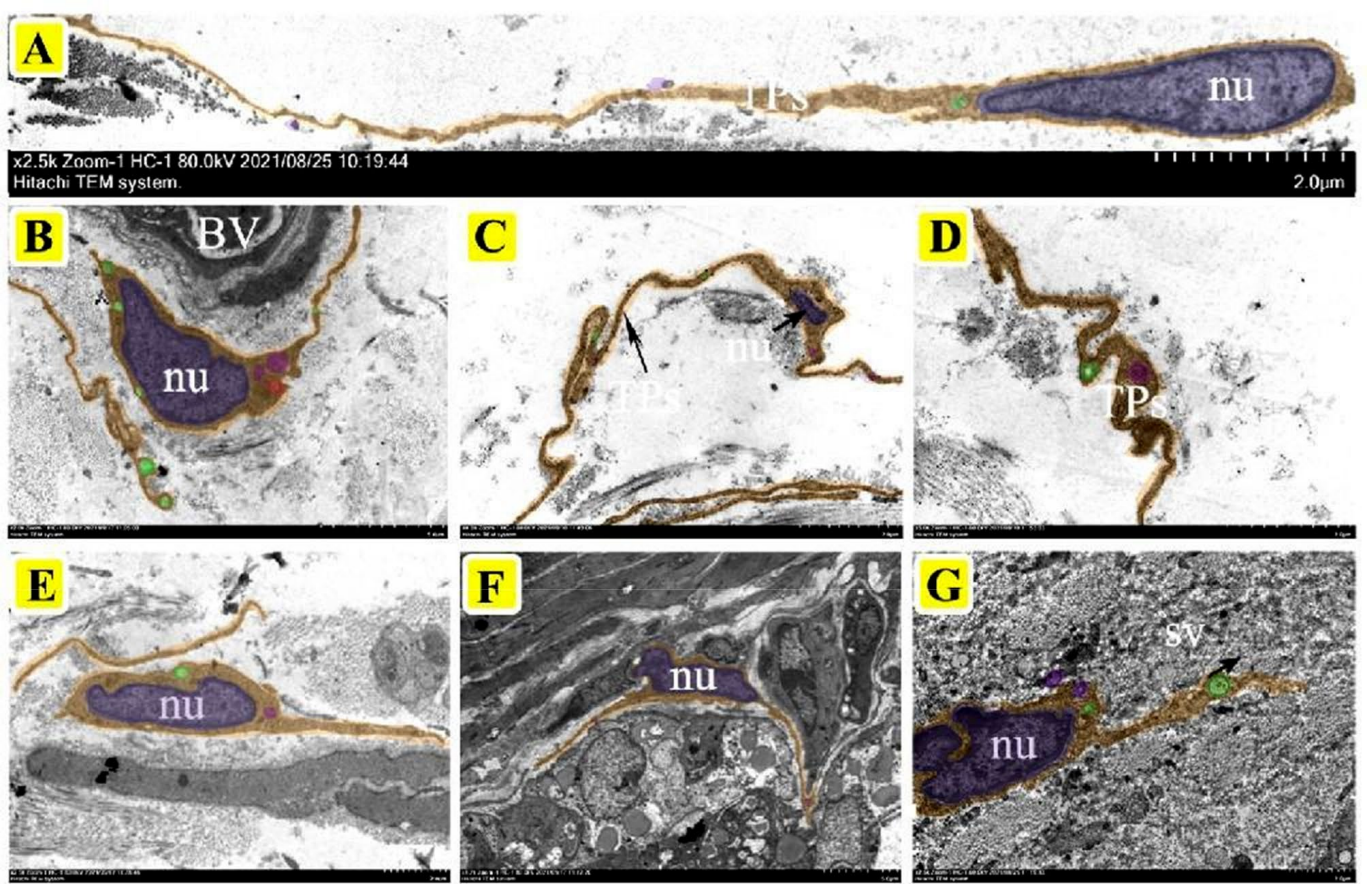
The localization of telocytes in the cauda epididymis of yak by TEM. (**A**, **B**) pear-shaped TCs occupied by a large nucleus around with secretory vesicles, mitochondria, lysosomes (Scale bar = 5 µm). (**C**, **D**) The TCs distributed outside the peritubular connective tissue had TPs closely associated with peritubular myoid cells and fibroblasts (Scale bar = 2 µm). (**E**) The TCs were composed of serrated nuclei and long TPs with secretory vesicles in the cells (Scale bar = 2 µm). (**F**) The TCs were close to the basement membrane and tightly connected to multiple epithelial cells by TPs forming a network (Scale bar = 5 µm). (**G**) TCs with long and curled TPs, irregular nuclei, and secretory vesicles (Scale bar = 2 µm). BV: blood vessel, nu: nucleus, SV: secretory vesicles.

TEM is the most effective method to identify TCs. TEM observation showed that the TCs in the epididymis of yak contained a large nucleus of indefinite shape; the most typical were ellipsoid, serrated, and pear-shaped. The nucleus, with obvious chromatin, was surrounded by a small amount of cytoplasm, rich in secretory vesicles and mitochondria. There were a large number of TPs composed of long cytoplasmic fragments (Fig. 1A,B). Most of the TCs in the epididymis of yak are distributed around the blood vessels and can be clearly observed to contact the blood vessels through TPs and may form special cell connections (Fig. 1C,D,E, and H). In addition, some of the TCs distributed in the interstitium of the epididymis were in contact with peritubular myoid cells and fibroblasts (Fig. 1F,G). The morphology of TCs in the corpus epididymis was similar to that in caput epididymis, which is characterized by a large body, thick cytoplasm, full nucleus, clear nucleolus, and with abundant secretory vesicles distributed around the nucleus of TCs (Fig. 2A–F). The nucleoli of TCs were clearly visible at high magnification and were also rich in extranuclear secretory vesicles and rough endoplasmic reticulum in Fig. 2G. Furthermore, there was also a slight difference in morphology between TCs distributed near the basal membrane and stroma in the cauda (Fig. 3A–G). TCs in the stroma had more plump cells, longer TPs, and numerous secretory vesicles, while the TCs outside the basement membrane had smaller bodies, larger nuclei, irregular strip shapes, and shorter TPs, they were also closely connected with many epithelial cells (Fig. 3A, B, and F). TPs as a signal communication tool extend to a variety of cells, forming a special network structure (Fig. 3B and F).

### Morphological structure of yak epididymis TCs under SEM

**Figure 4:**
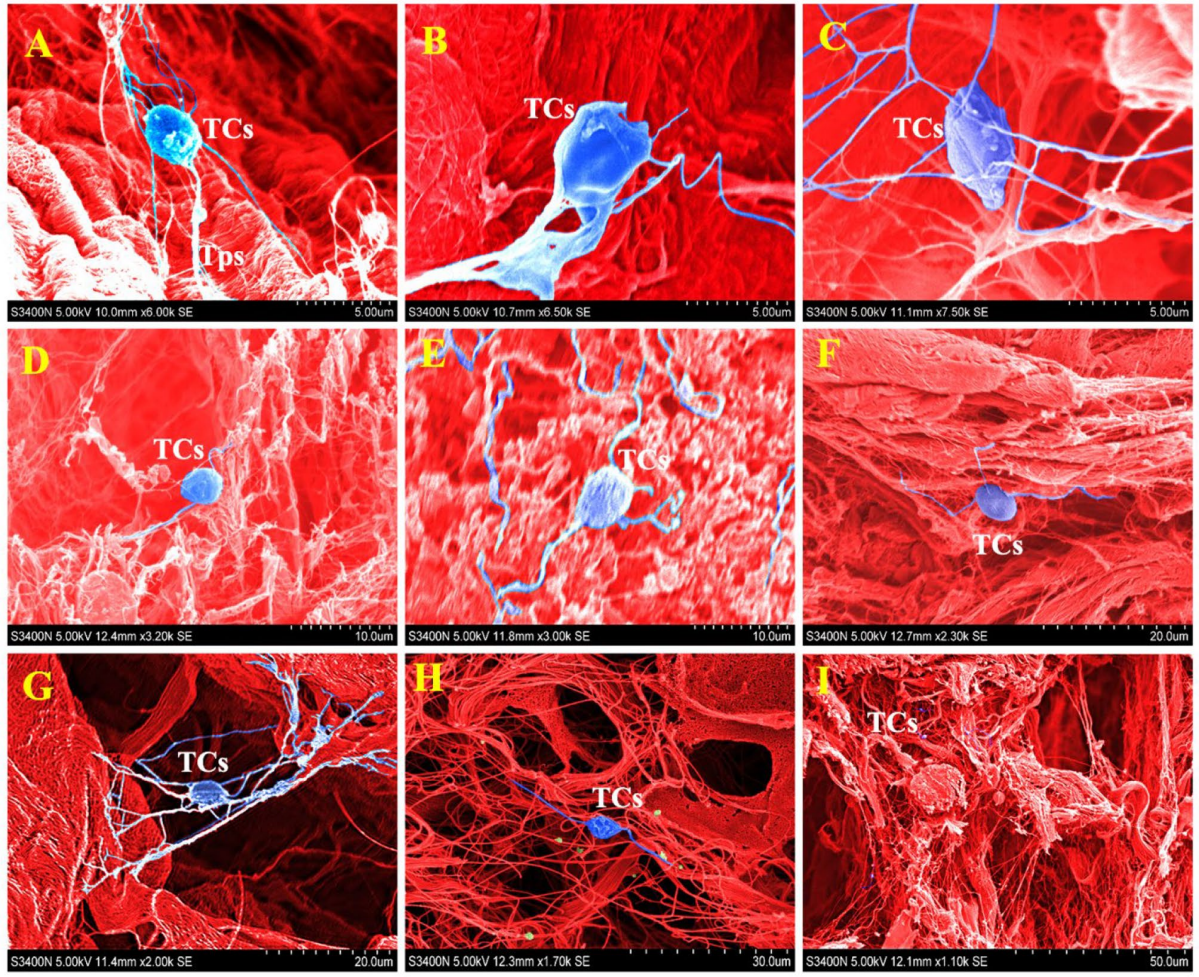
The localization of telocytes in the epididymis of yak by SEM. (**A**) The TCs were found in the epididymis of yak. There is a cytoplasmic projection structure in TPs, which has the typical characteristics of TCs cells. (**B**) TCs near the epididymis epithelium. (**C**) TCs in the interstitium of the epididymis. (**D**, **E**) TCs found in the stroma of the corpus epididymis. (**F**) TCs attached to the epididymis epithelium. (**G**) TCs form a network between the adjacent epitheliums. (**H**) The TCs in the interstitium of the epididymis cauda, with cell secretions were seen around them. (**I**) The cell population of TCs was found in the epididymis cauda.

The SEM photographs were colored using Adobe Photoshop 2020 software. It was found that TCs had complete cell morphology with the presence of obvious cytoplasmic processes on TPs. TCs usually attached to the epithelium or connected to other cells through TPs, but exist alone in the epididymis stroma. Moreover, the vast interactions can be used to make a complex TPs network between adjacent epithelial cells, and cell secretions are attached to TPs (Fig. 4A–I).

### Morphological model of telocytes

**Figure 5:**
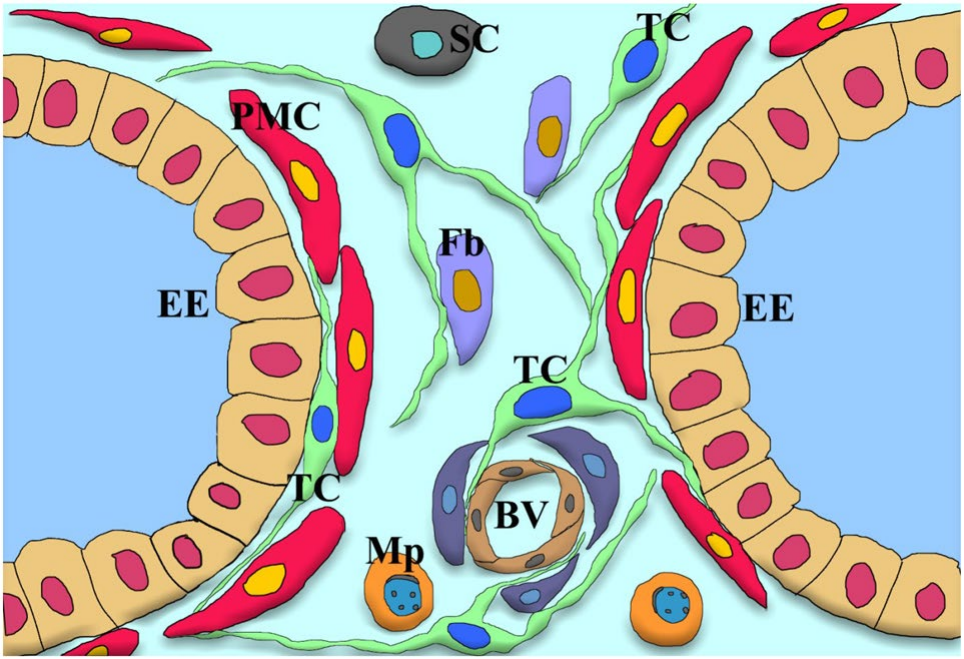
The distribution pattern of TCs in the yak epididymis based on the TCs found in this study. The green image shows that TCs are distributed between the epithelial cells (EE) and peritubular myoid cells (PMC), or between the fibroblasts (Fb) and vascular endothelial cells (VEC), and also attached to stem cells (SC) and macrophages (Mp).

**Figure 6:**
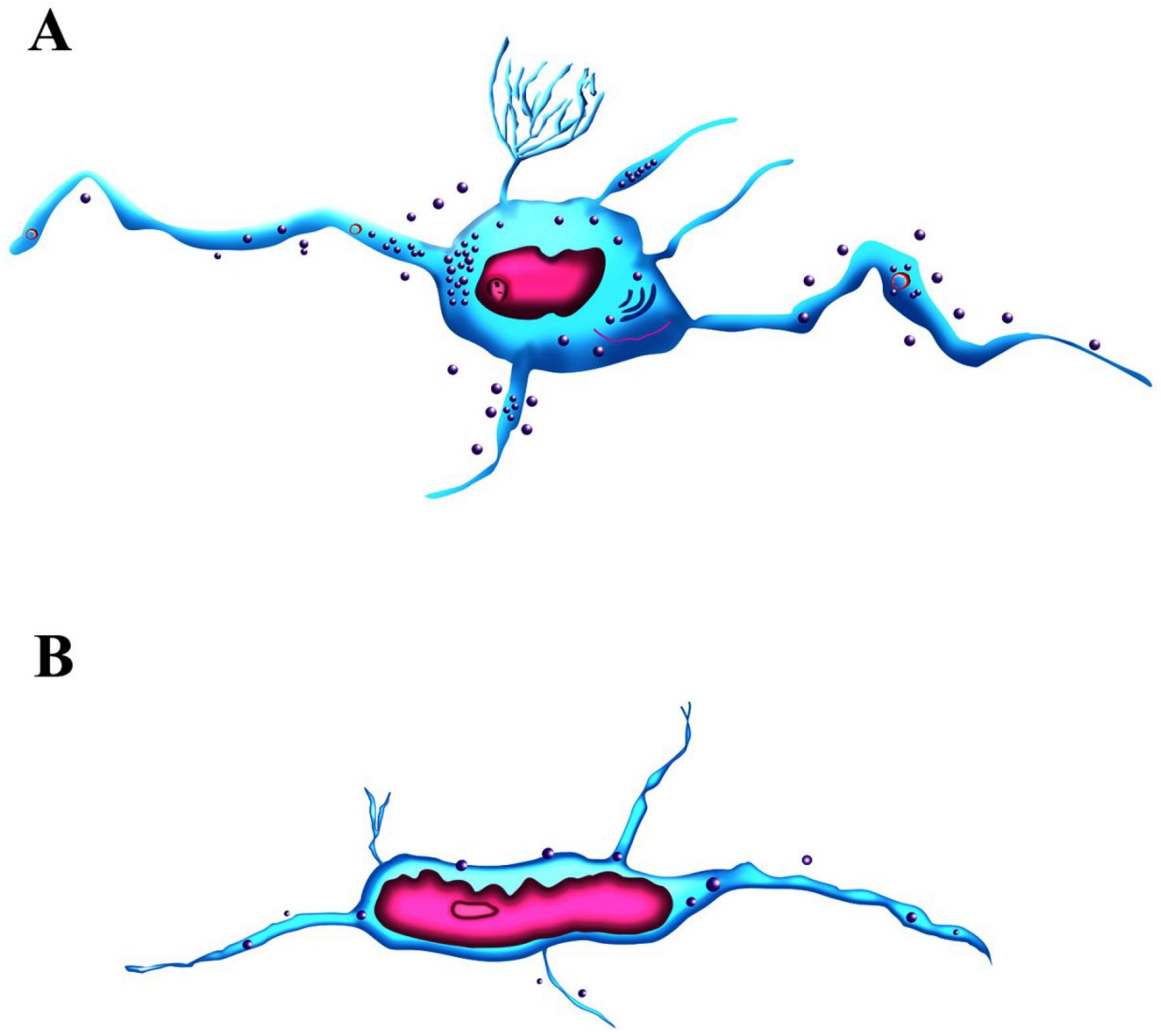
The morphology of TCs in the yak epididymis. (**A**) The TCs in the epididymis stroma. The round cell, obvious nucleolus, and slim and varied number of telopodes are the main characteristics of TCs. A large number of secretory vesicles, as well as mitochondria, and occasionally rough endoplasmic reticula are abundant in the cytoplasm. The exocytosis secretions are communicated by TPs. (**B**) The morphology of TCs close to the basal membrane of the epididymis tube. The small nucleus, irregularly long striped telopodes are the main feature of this type of TCs, but few secretory vesicles were distributed in the TPs. These cells may establish special connections with different cells of the epididymis epithelium.

The morphological structure and the morphological model diagram of TCs in yak epididymis were proposed based on the results of TEM and SEM (Figs. 5 and 6).

### TCs special staining results

**Figure 7:**
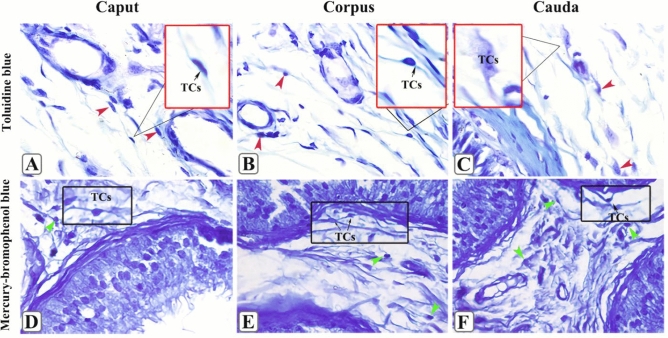
The toluidine blue and mercury–bromophenol blue staining of yak epididymis. Toluidine blue staining results of yak caput epididymis (**A**), corpus epididymis (**B**), and cauda epididymis (**C**). The distribution of TCs in the interstitium of epididymis surrounds the capillaries. The red arrow refers to telocytes, which are clearly visible. The photographs of mercury–Bromophenol blue staining in yak caput epididymis (**D**), corpus epididymis (**E**), and cauda epididymis (**F**). The cytoskeleton and TPs of TCs were stained dark blue by bromomercury phenol blue, and the green arrow indicates TCs. The morphology was clearly visible.

The toluidine blue staining results showed that a few TCs were distributed in the interstitium and more were distributed near the microvascular of the epididymis. However, they had different structures, adopting ellipsoid, spindle, pear, or other forms (Fig. 7A–C). Mercury–bromophenol blue staining showed that TPs were more obvious and the cytoplasmic processes on TPs were stained dark blue (Fig. 7D–F).

### Immunohistochemical and immunofluorescence analysis of TC surface markers

**Figure 8:**
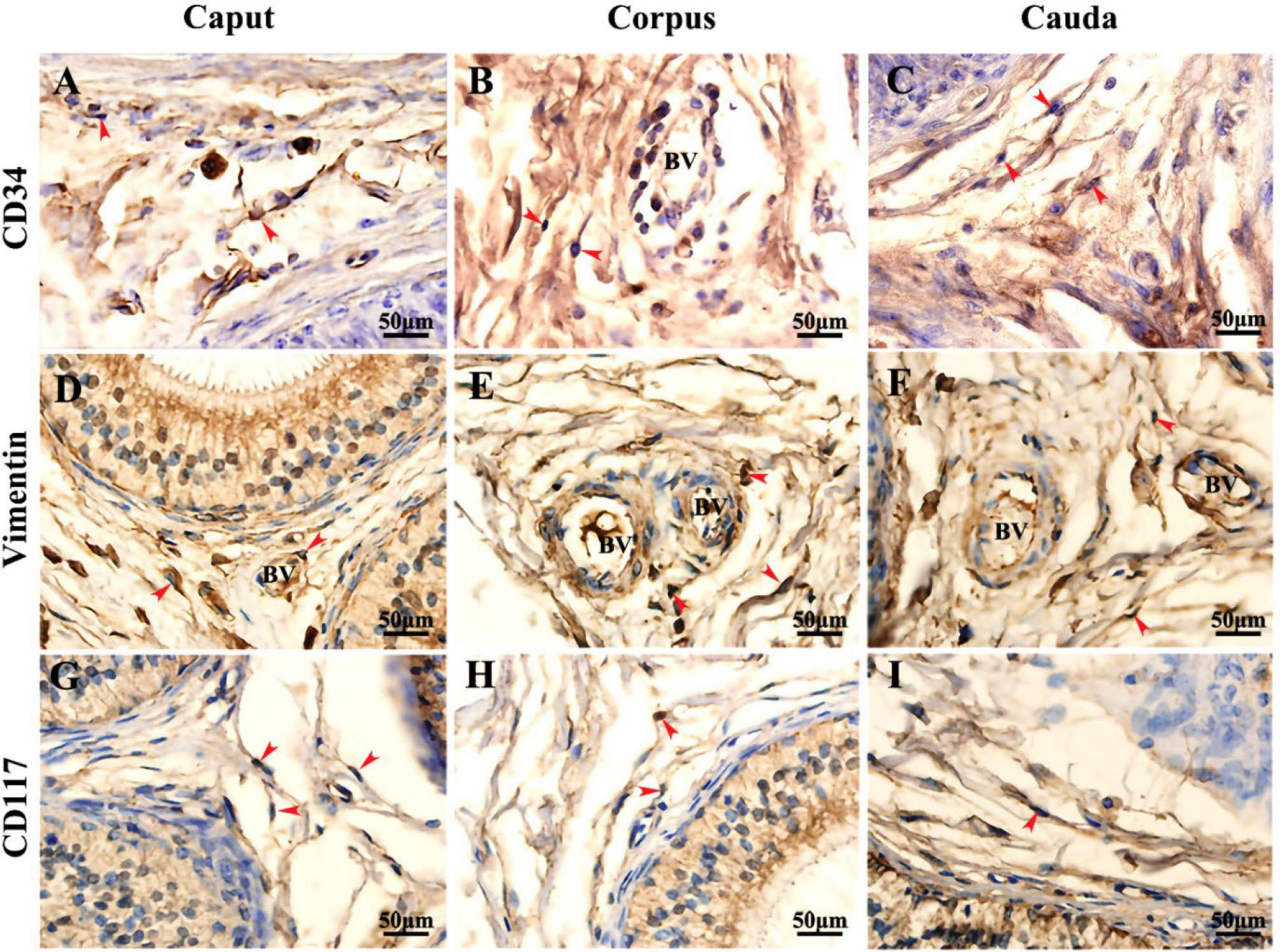
Immunohistochemical results of yak epididymis (Scale bar = 50 µm). (**A**–**C**) The immunohistochemical staining results of CD34 in caput, corpus and cauda of epididymis. (**D**–**F**) The immunohistochemical staining results of Vimentin in caput, corpus and cauda of epididymis. (**G**–**I**) The immunohistochemical staining results of CD117 in caput, corpus and cauda of epididymis. There were strong positive expressions of CD34, vimentin, and CD117 in the epididymal epithelium and epididymal stroma. Red arrows indicate TCs. BV: Blood vessel.

The immunohistochemical results showed that thick epithelial cells, mesenchymal cells, and capillaries were observed in the epididymis of yak. CD34 was strongly expressed in the interstitium and distributed mainly in the interstitium and near the epithelium (Fig. 8A–C). Compared with the caput and cauda, the CD34-positive intensity in the epididymis corpus was higher (Fig. 8B). Vimentin staining was strong positive in the caput, corpus, and cauda of yak epididymis. It is worth noting that vimentin has intensely positive expression in the stroma, epithelium, and microvascular wall of the epididymis (Fig. 8D–F). CD117 had strong positive expression in the caput, corpus, and cauda of yak epididymis, and the specificity was stronger than that of CD34 and vimentin. CD117 immunopositive cells were mainly distributed in the epithelial cytoplasm and mesenchymal cells (Fig. 8G–I).

**Figure 9:**
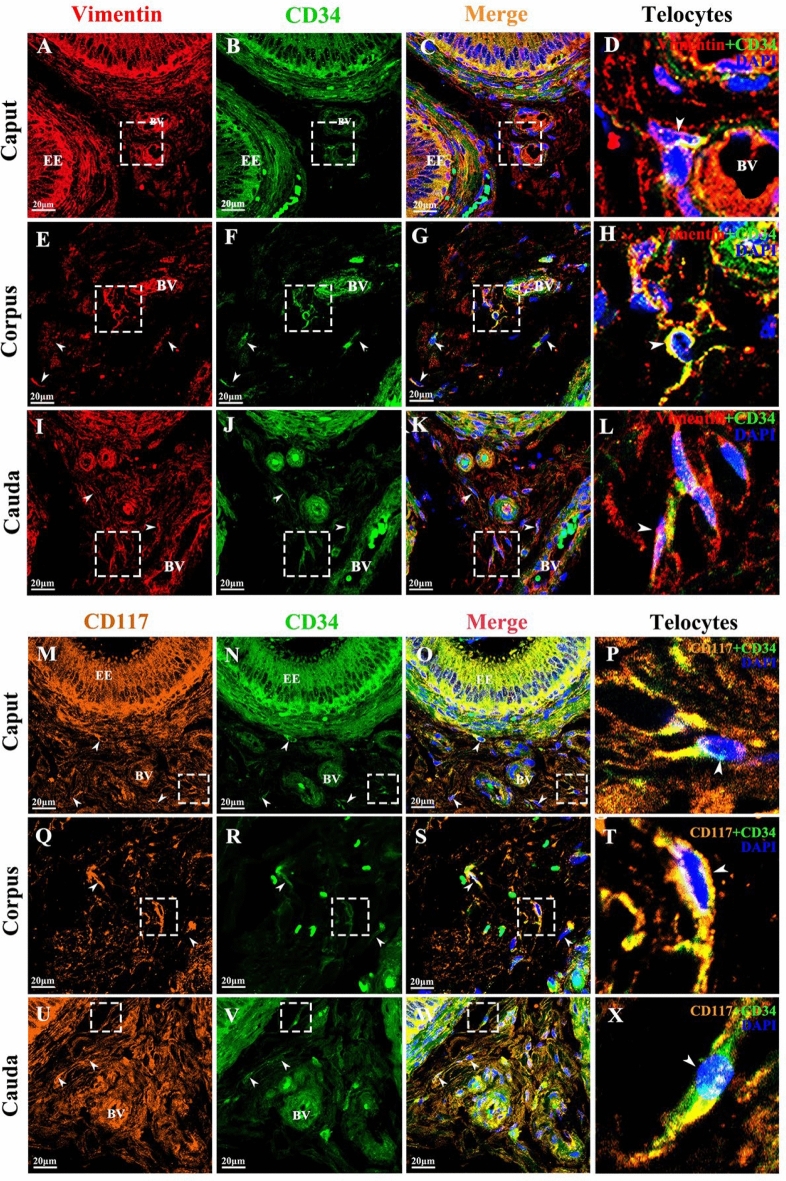
Double immunofluorescence results of yak epididymis staining for vimentin + CD34 and CD117 + CD34 (Scale bar = 20 μm). (**A**–**L**) Strong fluorescence expression of vimentin and CD34 in the epididymal epithelium and stroma. The co-expression of vimentin + CD34 was found around the capillary of the epididymal stroma. (**M**–**X**) Strong fluorescence expression of CD117 and CD34 in the epididymal epithelium and stroma. The co-expression of CD117 + CD34 was found around the capillary of the epididymal stroma. The co-expression position, shown by white arrows, denotes TCs. EE: Epididymal epithelium, BV: Blood vessel.

The immunofluorescence results showed that vimentin was widely expressed in the epididymis of yak, including fibroblasts, perivascular muscle-like cells and vascular endothelial cells, with strong positive expression (Fig. 9). These cells are present in the loose connective tissue around the tubulointerstitium. CD34 was positively distributed in the stroma and epithelium (Fig. 9). The CD34-positive oval cells near the epithelium were of a short shape, with cytoplasmic processes, which may be dendritic cells or TCs, and the positive expression was stronger in the caput. The co-expression of vimentin/CD34 was observed in the interstitium, capillary, and epithelium of yak epididymis (Fig. 9). There was strong positive expression of CD117 in the stroma and epithelium, with a large number of CD117-positive cells around the blood vessels of mesenchymal hair cells, and relatively few in the epithelium (Fig. 9). The cells with strong positive CD34 expression and weakly positive CD117 expression appeared in the yak epididymis corpus; they may be phagocytes or lymphocytes (Fig. 9). CD117/CD34 co-expression was observed in the interstitial epididymis, as shown by yellow fluorescence (Fig. 9). The common features were proved by the co-expression of vimentin/CD34 and CD117/CD34 and showed that the cells had a long cytoplasmic extension and a large nucleus, and that most of them were distributed around the interstitial capillaries of yak epididymis and outside the epididymal epithelium. The obtained morphology conformed to the basic characteristics expected for TCs.

### The mRNA and protein expression of effective markers of telocytes in yak epididymis

**Figure 10:**
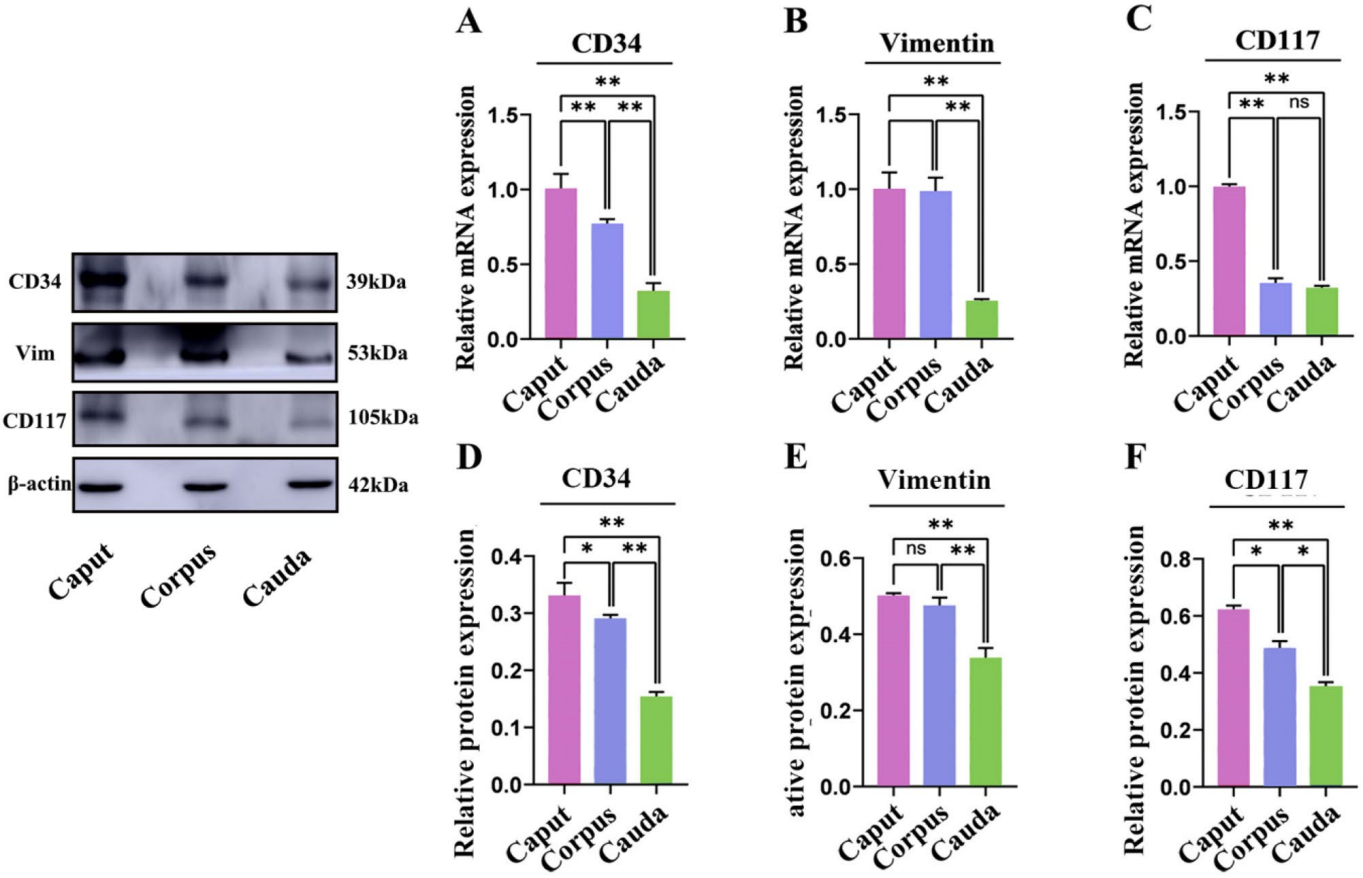
mRNA and protein expression of effective telocyte markers (CD34, vimentin, CD117) in the epididymis of yak. Each sample was tested three times. Results are expressed as the mean ± standard deviation, with β-actin gene and protein expression used as an internal control. ** *p* < 0.01; * *p* < 0.05; NS (not significant), *p* > 0.05.

The expression of CD34, Vimentin, and CD117 mRNA in yak epididymis was detected by qRT-PCR. The expression of CD34 mRNA in caput epididymis tissues of yaks compared to corpus and cauda showed an elevated trend (*p* < 0.01). Although there was no significant difference in the expression trend in vimentin mRNA in caput and corpus, it was significantly higher than that in the cauda epididymis (*p* < 0.01). Similarly, CD117 mRNA expression in yak caput epididymis was also significantly higher than that in the corpus and cauda (*p* < 0.01) (Fig. 10). Western blotting results showed that the expression levels of the proteins of CD34, vimentin, and CD117 in the caput epididymis of yak were significantly higher than those in the corpus and cauda (Fig. 10); moreover, protein expression trends patterns were consistent with mRNA expression. In summary, the relatively high gene transcription and protein translation levels of TC surface markers in the caput epididymis of yaks were probably closely related to its physiological function.

## Discussion

Homeostasis of the epididymal microenvironment guarantees male animal reproductive ability. As a type of interstitial cell, TCs play an important role in the epididymis immune microenvironment homeostasis and blood–epididymis barrier function. TCs have been found in many animal tissues, such as the human colon^41^, fish brain^16^, and poultry skin, and TCs have common features: slender cytoplasmic extensions and a large nucleus. In addition, TCs were also found in the testis of rats^42^, rabbits^43^, and camels^44^. TCs found in our study have common morphological characteristics with previous studies. The large nucleus is one of the features of TCs, and serrated nuclei of TCs have so far been described only in camel testes, whereas the same was present in the serrated nuclei of the TCs in yak cauda.

TEM has been considered to be the most effective way to differentiate TCs from other mesenchymal and epithelial cells^45^. In this study, the TEM ultrastructure of TCs in different locations of the yak epididymis were identified. The TCs distributed around the capillaries or near the basement membrane had differences in morphology. The former has more full cell bodies and TPs are relatively long, whereas the latter had contrasting features. Some scholars have confirmed that TCs around the capillaries may be associated with angiogenesis and material exchange^46^. TCs near the epididymis basement membrane were closely connected with the peritubular myoid cells, which provide structural support for the epididymal duct, and may participate in the contraction of the epididymal duct, providing an impetus for the transport of sperm. The TEM analysis of the ultrastructure revealed that there were multitudinous secretory vesicles in the TPs of yak epididymis TCs; these were released to the surrounding environment of TCs in the form of exocytosis and were easily observed by TEM.

Our findings show that secretory vesicles also exist in TCs found in the human testis, myocardium, ovary, and other tissues. Considered the “messengers” of TCs that communicate with the outside world^47^, this feature is regarded as one of the most important conditions for distinguishing TCs from other interstitial cells in ultrastructure. We found TCs in the interstitium of the yak caput epididymis by SEM were mainly distributed in the interstitium and around the blood vessels. The differently sized TPs from TC cell bodies, as well as the network structure composed of dense TPs, can be observed by SEM, which is also one of the important characteristics of TCs.

In this study, we explored multiple special staining methods and found that mercury–bromophenol blue stained TPs and the cytoplasmic processes dark blue. The cytoplasmic processes of TCs have the function of secreted proteins, and TCs in the yak epididymis of during the estrous season may be involved in the reproductive regulation of the epididymis through the secretion of related proteins by the cytoplasmic processes of TPs; thus, this suggests the need for further study of the seasonal variation characteristics of TCs.

Studies have shown that immunohistochemistry can be used as a basic method to localize TCs^48^. Because there was strong expression of vimentin in the stroma and epithelium of epididymis, the immunohistochemistry and double immunofluorescence co-localization were designed to determine the location of TCs. We found that the cells with double-positive expression of vimentin/CD34 and CD117/CD34 were similar to the TCs phenotype in the stroma and capillary of yak epididymis. These cells had a long cytoplasmic extension and oval nuclei, which were consistent with the basic characteristics of TCs. Similar results were found in testis of humans^47^, dove^49^, and Pelodiscus sinensis^50^. We found that the mRNA and protein expression of CD34, vimentin, and CD117 were relatively high in the yak caput epididymis. The caput epididymis plays an important role in sperm maturation and processing, especially in sperm concentration, maturation, and transport^51,52^. Compared with the corpus and cauda, the caput is more like a sperm “processing workshop”, suggesting that high expression of TCs markers in the epididymis caput may be related to sperm processing.

Via double immunofluorescence staining, we also found that TCs were associated with dendritic cells, peritubular myoid cells, and lymphocytes through TPs. We believe that there is a specific network structure between TCs and the epithelium and stroma in the epididymis of yak, namely the TCs network. Following a previous discovery that the network structure of TCs in human testis may be related to the blood–testicular barrier^48^, the TCs network of yak epididymis TCs may play an important role in the formation of yak blood–testicular barrier. Studies have found that TCs present in the epididymis of camels are positive for vascular endothelial growth factors^53^, indicating that TCs are probably involved in angiogenesis in the epididymis of the camel. TCs promote angiogenesis by secreting extracellular vesicles containing microRNA^54,55^. Angiogenesis is a process by which monolayer endothelial cells (ECs) control the permeability of blood cells through material exchange, and maintain homeostasis of the vascular environment and a series of physiological phenomena leading to the formation of new blood vessels^56^. Microvessels in the epididymis are considered to be the prerequisite for sperm maturation and transport^57^. Interestingly, we found that TPs of TCs were always extremely close to the blood vessel and extended to the vascular wall. Combined with TEM evidence, we speculated that TCs may be a material exchange pump that play an important role in the process of material and energy exchange between interstitial and nutrient vessels in the epididymis.

At present, more function of TCs are emerging^58,59^, such as injury repair^60,61^, vascular regeneration^62^, and cell communication^63^. However, there are few studies of TCs in animal reproduction. Studies have found that the secretory vesicles of TCs in camel testis are affected by seasonal changes, with more secretion in spring and less secretion in summer^44^. There is some relationship between TCs secretion vesicles and camel estrus. However, there is insufficient evidence to prove TCs are involved with the regulation of animal estrus. Some researchers believe that TCs may indirectly affect the secretion and release of androgen by establishing intercellular connections with other interstitial cells to regulate the reproductive activities of male animals^48^. Furthermore, studies reported that TCs express progesterone and estrogen receptors in the female gonadal axis^64–66^. The emergence of such studies is constantly drawing TCs into the field of reproductive study. Our study revealed the morphological structure and distribution location of TCs in yak reproductive organs and provided a reference for the study of TCs in animal reproduction in a hypoxic plateau environment.

## Methods

### Animals and sample acquisition

The epididymal tissue of adult healthy yak (n = 10; ≥ 3 years) was collected from designated slaughterhouses from July to August in Xining City, Qinghai Province (at an average altitude of 3100 m) China. Based on their anatomical characteristics, epididymis samples were divided into three parts: caput, corpus, and cauda. A portion of each sample was quickly frozen in liquid nitrogen, transported to the laboratory and then stored at −80°C for RNA and protein extraction; the remaining samples were stored in 4% paraformaldehyde and 2.5% glutaraldehyde separately for histological and ultrastructural study. All experimental animals were approved by the Animal Care and Use Committee of the Veterinary College of Gansu Agricultural University (Ratification number: GSAU-Eth-VMC-2021-010), and all methods were performed in accordance with the relevant guidelines and regulations.

### Drugs and reagents

All experimental antibodies were purchased from commercial suppliers. Rabbit polyclonal antibody CD34 (bs-8996R), vimentin (bs-8533R), and CD117 (bs-1005R) were purchased from Beijing BIOSS Antibodies Co., Ltd, China. Goat anti-rabbit IgG H&L (ab150077, Alexa Fluor® 488; ab150079, Alexa Fluor® 647; ab150080, Alexa Fluor® 594) were provided by Abcam,Cambridge, UK. The DAB color reagent kit (PA110) was provided by Beijing TIANGEN Biotechnology Co., Ltd. The immunohistochemical staining kit (SP-0023) used was produced by ZYMED USA, Beijing BIOSS Biotechnology Co., Ltd. ECL Plus ultrasensitive luminescent solution (PE0010) was purchased from Solebao Biotechnology Co., Ltd.

### Sample preparation and observation

Preparation of ordinary samples: Epididymal tissue samples (0.5 × 0.5 × 0.5 cm) were fixed with 4% paraformaldehyde solution and rinsed in running water for 24 h before gradient ethanol dehydration. Subsequently, samples were made transparent with xylene, embedded using an Epon 812 paraffin embedding machine, and 4-μm-thick serial sections were cut. Adjacent slices were used for toluidine blue staining, mercury–bromophenol blue staining, immunohistochemistry and immunofluorescence staining, respectively.

SEM sample preparation: The epididymal tissue of yak was cut into 0.2 cm × 0.2 cm × 0.2 cm pieces and fixed with 2.5% glutaraldehyde for 2 days. The tissue was washed four times with 0.1 mol/L phosphate buffer; each wash was 15 min. The samples were treated with 1% OsO4 for 1 h and washed with double-distilled water six times (10 min each wash). Then, the tissue was treated with 2% tannic acid for 30 min and washed with double-distilled water six times (each wash 10 min), and then subjected to gradient ethanol dehydration (30%, 50%, 70%, 80%, 90%, 95%, and 100%; each stage 30 min) and then soaked in isoamyl acetate for 30 min. Tissues were dried by critical point drying, and then the samples were sprayed with gold and observed using a scanning electron microscope.

TEM sample preparation: The epididymal tissue of yak fixed in 2.5% glutaraldehyde was cut into small pieces (0.2 cm × 0.2 cm × 0.2 cm) and fixed in 2% osmium tetraoxide at 4°C for 3 h. The pieces were dehydrated with a gradient acetone series (30%, 50%, 70%, 80%, 90%, 95%, and 100%) and then embedded in epoxy resin. Ultrathin sections were prepared and affixed to the copper mesh, stained with uranium acetate and lead citrate, and then examined using a JEM-100CX electron microscope (Japan NEC).

### Immunohistochemistry and immunofluorescence

The epididymal tissues were embedded in paraffin and cut into 4-µm-thick sections. Paraffin sections were dewaxed and dehydrated, repaired with microwave oven antigen retrieval, blocked with 3% H2O2 solution for 10 min, and incubated with goat serum albumin for 15 min. Subsequently, 50 µL rabbit polyclonal antibody (CD34, Vimentin, and CD117) diluted to 1:300 was added to each slide; the negative control consisted of 0.01 mol/L PBS instead of the first antibody. The slides were incubated at 37 °C for 4 h, washed three times with phosphate buffer solution (PBS) (each wash 5 min) and then 50 µL of biotin-labeled goat anti-rabbit IgG working solution was added and the sections were incubated at 37 °C for 15 min and washed three times with PBS (each wash 5 min). Horseradish enzyme-labeled streptavidin solution was added and washed with PBS three times (each wash, 5 min). The DAB color developing solution was added for 5–20 min. Hematoxylin counterstaining was performed for 5 min; then, sections were dehydrated by an alcohol gradient, made transparent with xylene, and sealed with neutral gum. The sections were observed under a microscope.

Immunofluorescence staining was performed with the primary antibody; sections were incubated at 37°C for 4 h and rinsed with PBS three times. Subsequent steps were completed in a dark room. Anti-Rabbit IgG H&L AF488 or AF594 (dilution ratio 1:1000) was added, incubated at 37°C for 1 h, and washed five times with PBS (each wash 5 min). Then, the second antibody was added and the sections were incubated at 37°C for 4 h, washed five times in PBS (each wash 5 min). Rabbit Anti-PHD2/AF647 (dilution ratio 1:1000) was added dropwise and incubated at 37°C for 1.5 h. After washing with PBS, DAPI was added dropwise and incubated in a dark room for 10 min. After further washing with PBS, the patch was sealed with a capping agent and the sections were observed under a laser confocal microscope. The negative control consisted of 0.01 mol/L PBS instead of the first antibody. The remaining conditions and steps were the same.

### qRT-PCR analysis

The caput, corpus, and cauda tissues of yak epididymis stored at − 80 °C were removed from storage, and 0.1 g was weighed and placed into a mortar. Liquid nitrogen was added to the grind, and 1 mL Transzol was added to the shock treatment. Then 0.2 mL of chloroform was used to extract RNA and confirm its purity. cDNA was synthesized by RNA reverse transcription, and stored in a refrigerator at − 80 °C for further use. Primer Premier 5.0 software was used (Primer Biosoft International, Palo Alto, USA) was used to design primers; the primer sequence was obtained with reference to the NCBI database (www.ncbi.nlm.nih.gov), and the primer information is shown in Table 1. The β-actin gene was used as the internal reference. qRT-PCR was performed using a Light Cycler 480 thermocycler (Roche, Mannheim, Germany) in a final reaction volume of 20 μL, comprising 1 μL of cDNA, 1 μL of forward primer, 1 μL of reverse primer, 10 μL of 2 × SYBR Green II PCR mix (TaKaRa, Dalian, China), 0.4 μL of ROX reference dye, and 6.6 μL of nuclease-free H2O. The cycling reaction conditions were 95 °C for 30 s; followed by 95 °C, for 5 s, and 60 °C for 30 s each, for a total of 45 cycles. Three replicates were performed for each sample to ensure relative expression accuracy of the target genes.

**Table 1:** Primer information.

Gene	Sequence (5′ → 3′)	Product length (bp)	Tm (°C)	Reaction efficiency (%)	Accession No
CD34	CTGCTGAGTCTGCTGCCTTCT	189	59	101	AB021662.1
	GCTGTGGTCCCATTGCTGT				
Vimentin	GGATGTTTCCAAGCCTGAC	139	58	98	NM_173969.3
	GGCATCATTGTTGCGGTTA				
CD117	ACAATGGGACGGTGGAGTG	122	60	94	D16680.1
	GTTGCCGCACTTGTCCCAC				
β-Actin	ACGGTGCCCATCTACGAGG	153	60	104	DQ838049.1
	CTTGATGTCACGGACGATTT				

### Western blotting analysis

From the epididymal tissue of yak stored at − 80 °C, 0.1 g was collected and placed into a mortar. Liquid nitrogen was added and the tissue was ground into a fine powder with a pestle. Then, protein cracking liquid was added and samples were cracked on ice for 3 h after eddy shock. Tissue and cell lysates were centrifuged at 12,000 rpm at 4 °C for 15 min and the supernatant was stored at − 80 °C. Determination of protein concentration by BCA protein assay kit (PC0020, Solarbio Biotechnology Co., Ltd., Beijing, China), and all proteins were diluted to the same concentration. Protein samples (25 μg) were separated by SDS–polyacrylamide gel electrophoresis (SDS-PAGE) using a 5% stacking gel and 12% separating gel. After electrophoresis, the separation gel was cut according to the size of the target protein and referred to the Marker, and the cut target bands were then wet-transferred to the support membrane, and the membrane was incubated with primary antibodies (1:800) at 4 °C overnight and washed with Tris-buffered saline + Tween 20 (TBST). Horseradish peroxidase-labeled goat anti-rabbit IgG was used as the secondary antibody and the incubation was performed for 2 h at 37 °C in TBST buffer for 10 min. The polyvinylidene fluoride membrane was then subjected to chemiluminescence detection. Chemiluminescent substrate solutions A and B were mixed at a ratio of 1:1, and the reaction proceeded at 25 °C. The transfer membrane was photographed for analysis, with β-actin used as the internal reference.

### Statistical analysis

Western blotting data were quantified by ImageJ software (National Institutes of Health, Maryland, USA). The qRT-PCR data were analyzed by the 2 − ΔΔCT method; the obtained results were subjected to the dominance test by SPSS 17.0 statistical software, and the histogram was plotted by GraphPad 9.0 software.

### Institutional review board statement

The study is reported in accordance with ARRIVE guidelines. And all experimental animals were approved by the Animal Care and Use Committee of the Veterinary College of Gansu Agricultural University (Ratification number: GSAU-Eth-VMC-2020-016w).

## Supplementary Information

Supplementary Information 1.

Supplementary Information 2.

## Data Availability

The datasets used and/or analysed during the current study available from the corresponding author on reasonable request.
