# Supplementary material for: Morphological and histochemical identification of telocytes in adult yak epididymis
Source: Sci Rep. 2023 Mar 31;13:5295. doi: 10.1038/s41598-023-32220-4 (PMC10066225; doi:10.1038/s41598-023-32220-4)
Supplement: Supplementary file 1 — Supplementary Information 1. [file 41598_2023_32220_MOESM1_ESM.doc]

**Original Images for Gels**


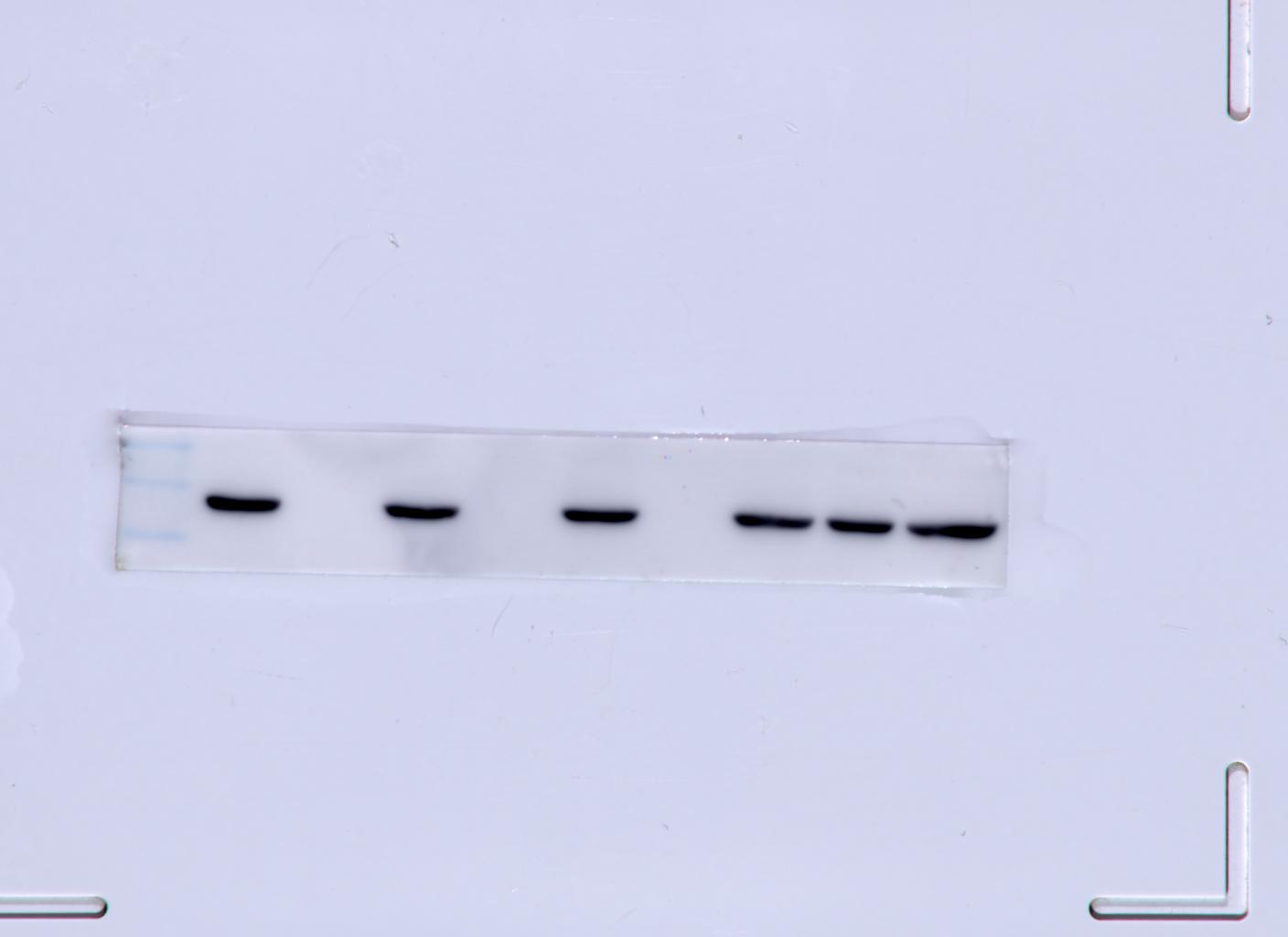


β-actin

A1

42KDa

35KDa

48KDa

Caput

Corpus

Cauda

β-actin


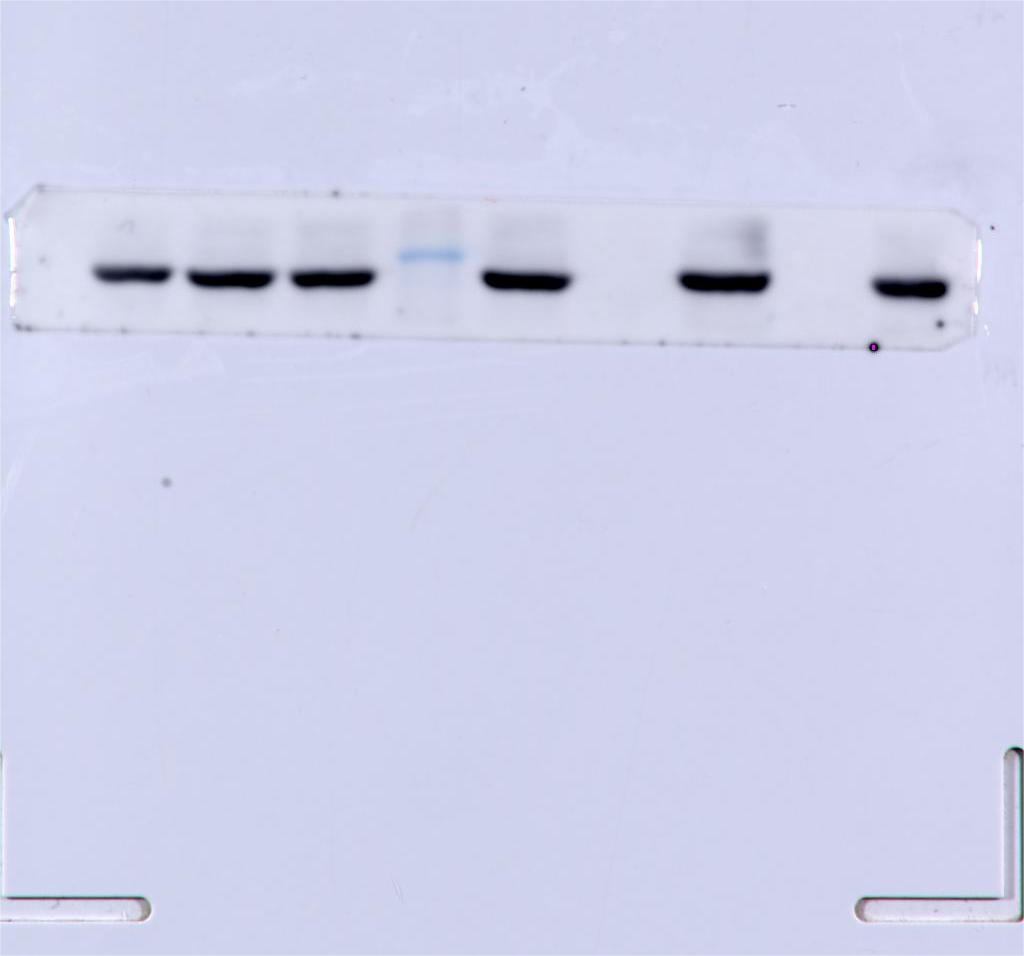


Caput

Corpus

Cauda

42KDa

48KDa

A2


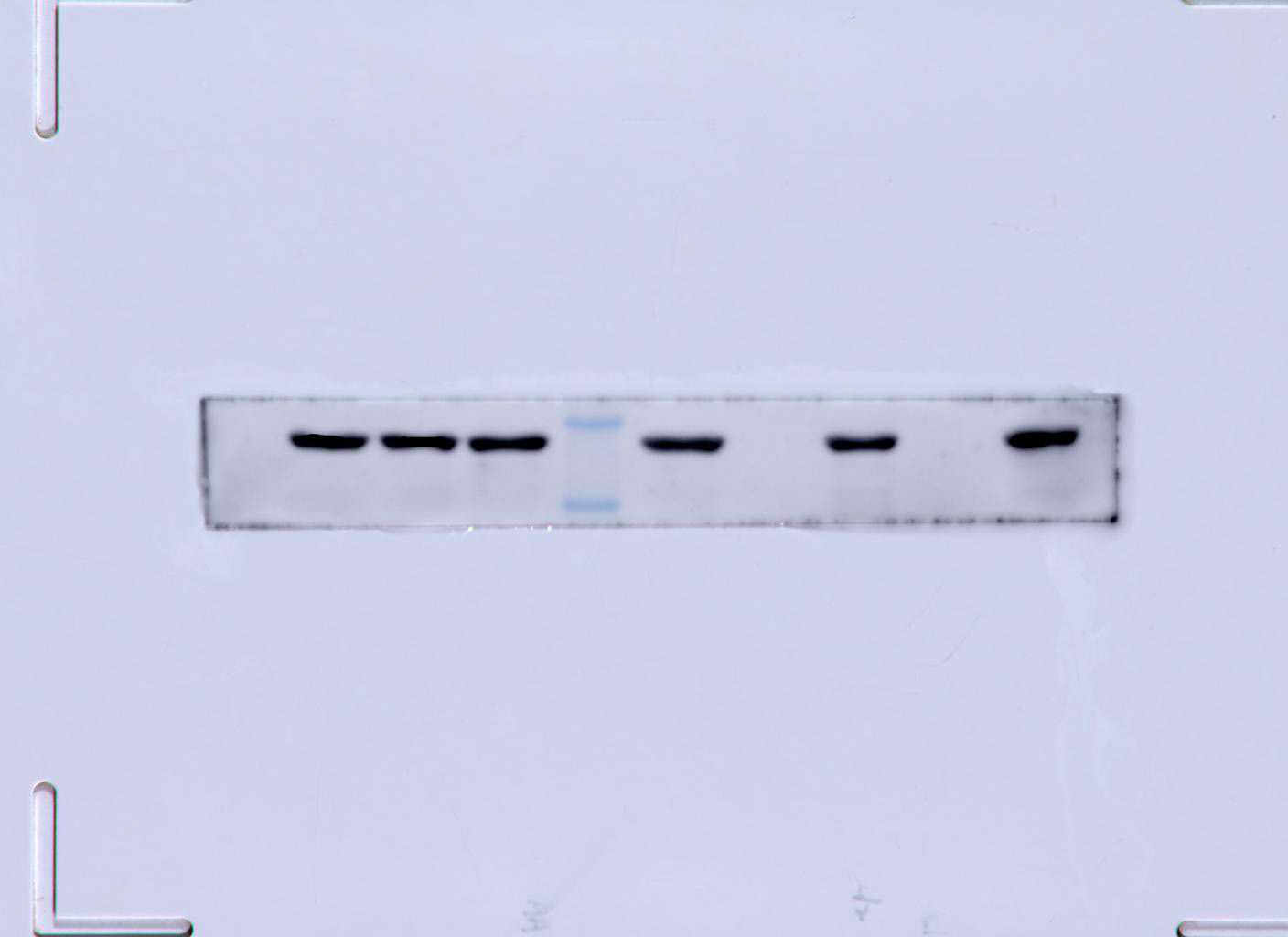


Cauda

Caput

Corpus

β-actin

A3

42KDa

48KDa

35KDa

**Figure A1, A2 and A3** are the gel images of three replicate experiments of β-actin Western blot in the caput, corpus and cauda of yak epididymis. All Western blot gels are cropped with reference to the Marker prior to hybridization with the antibody. Gel strips are developed in a chemiluminescence instrument. The red box indicates the target protein. The blue print is Marker. The same below.

B1


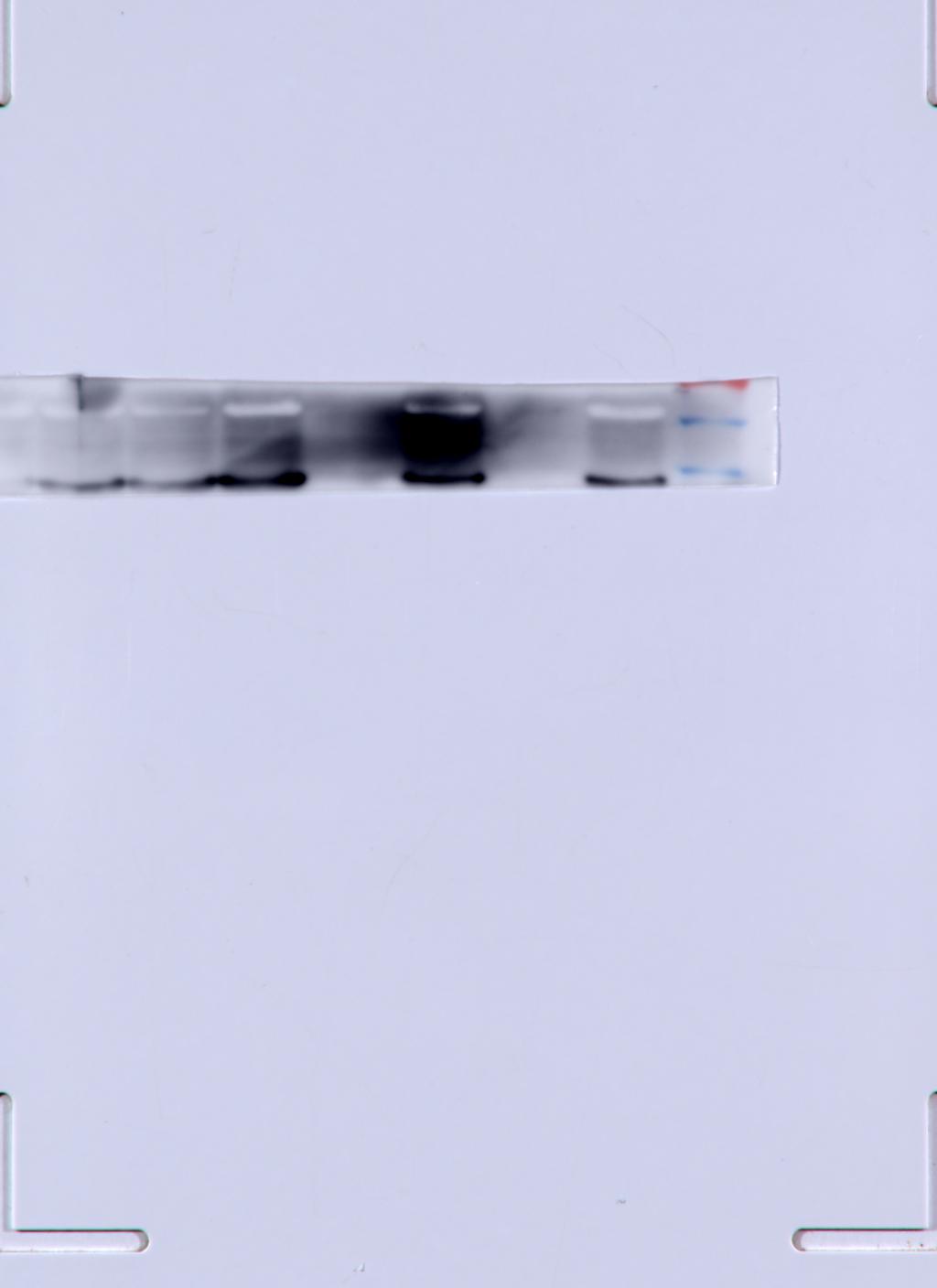


Caput

Cauda

Vimentin

Corpus

53KDa

63KDa


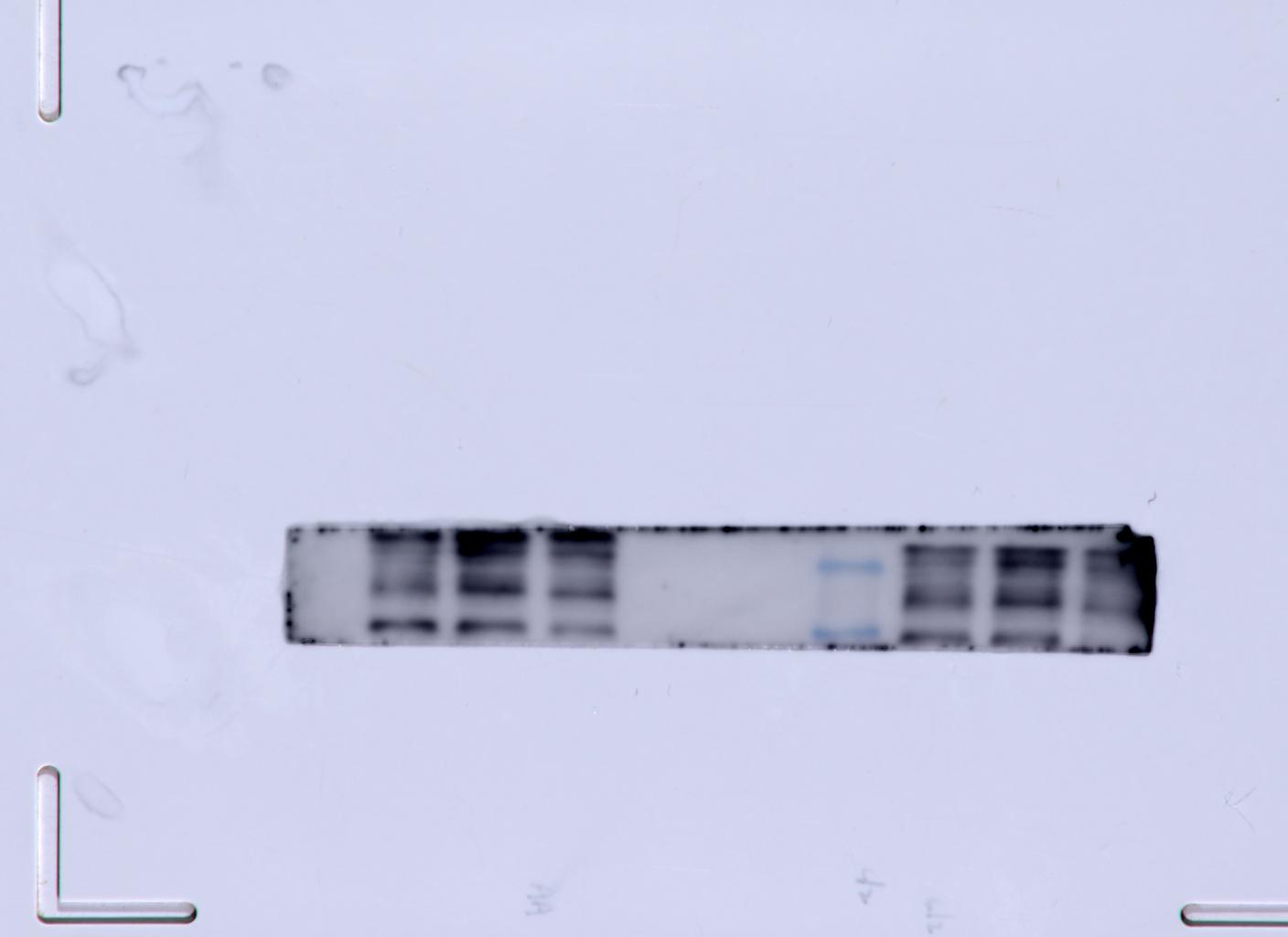

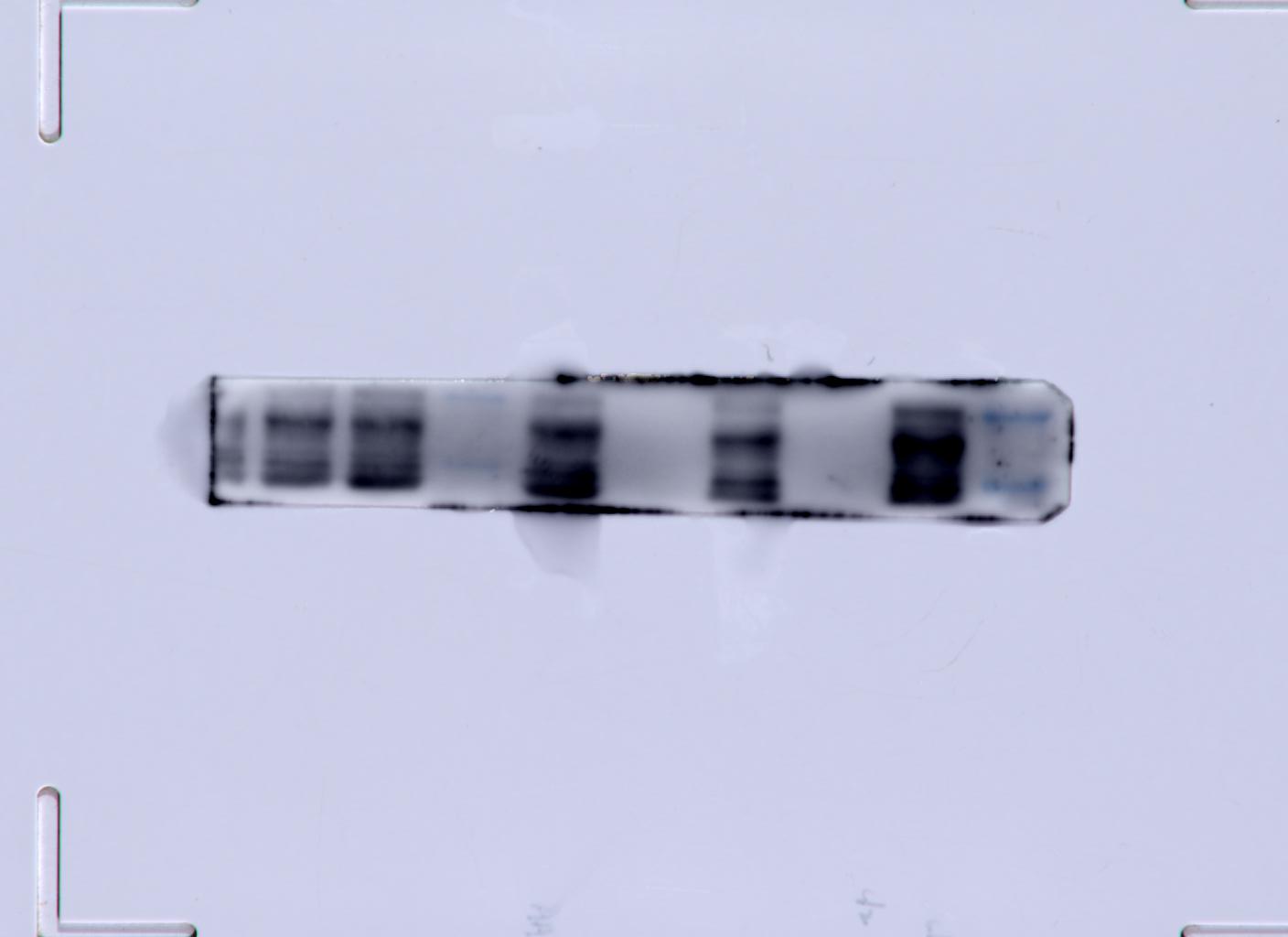


Corpus

Caput

Corpus

Caput

Cauda

Cauda

Vimentin

Vimentin

B3

B2

63KDa

48KDa

53KDa

48KDa

63KDa

53KDa

**Figure B1, B2 and B3** are the gel images of three replicate experiments of Vimentin Western blot in the caput, corpus and cauda of yak epididymis.


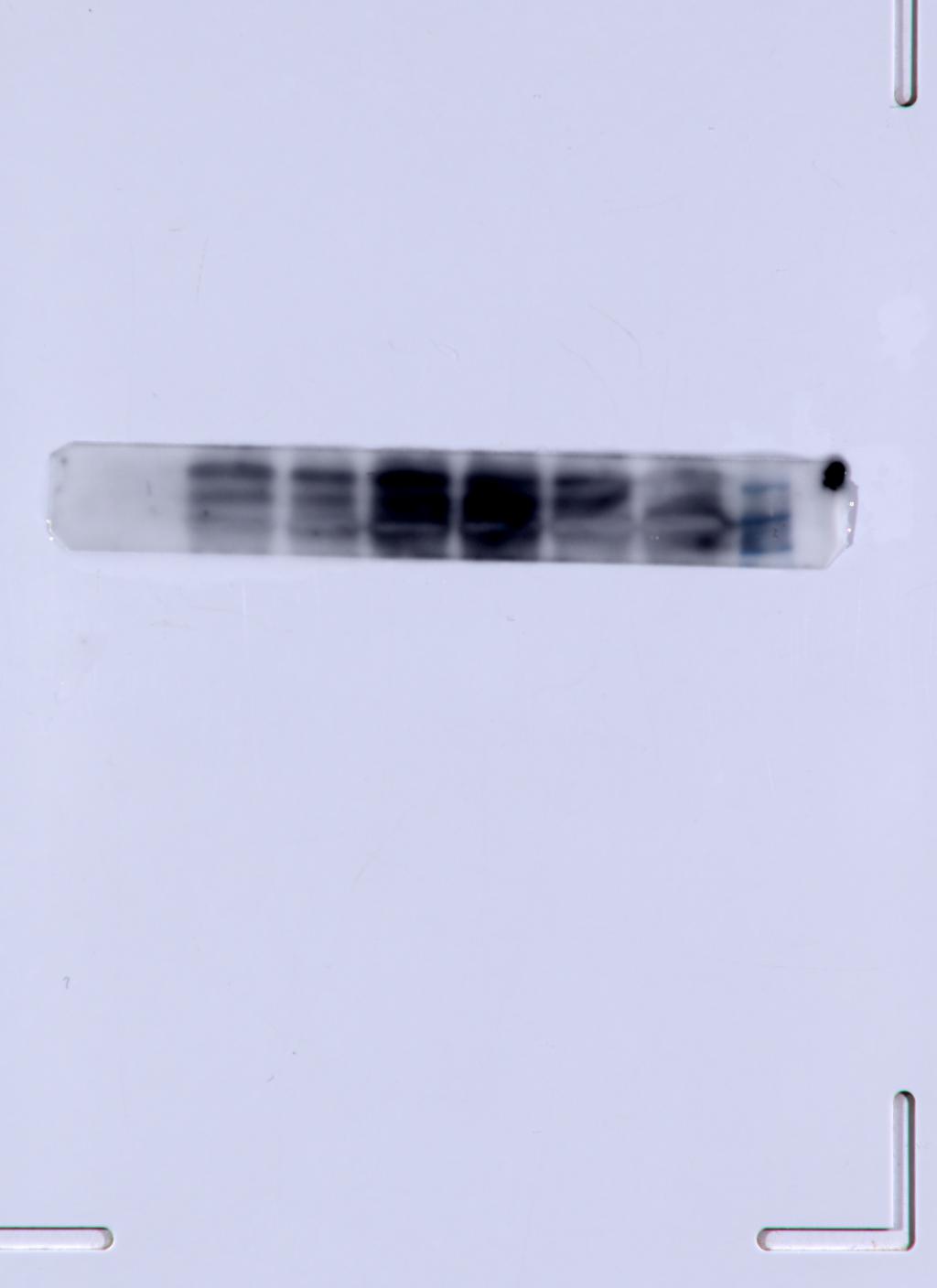


CD34

C1

39KDa

48KDa

35KDa

Caput

Corpus

Cauda


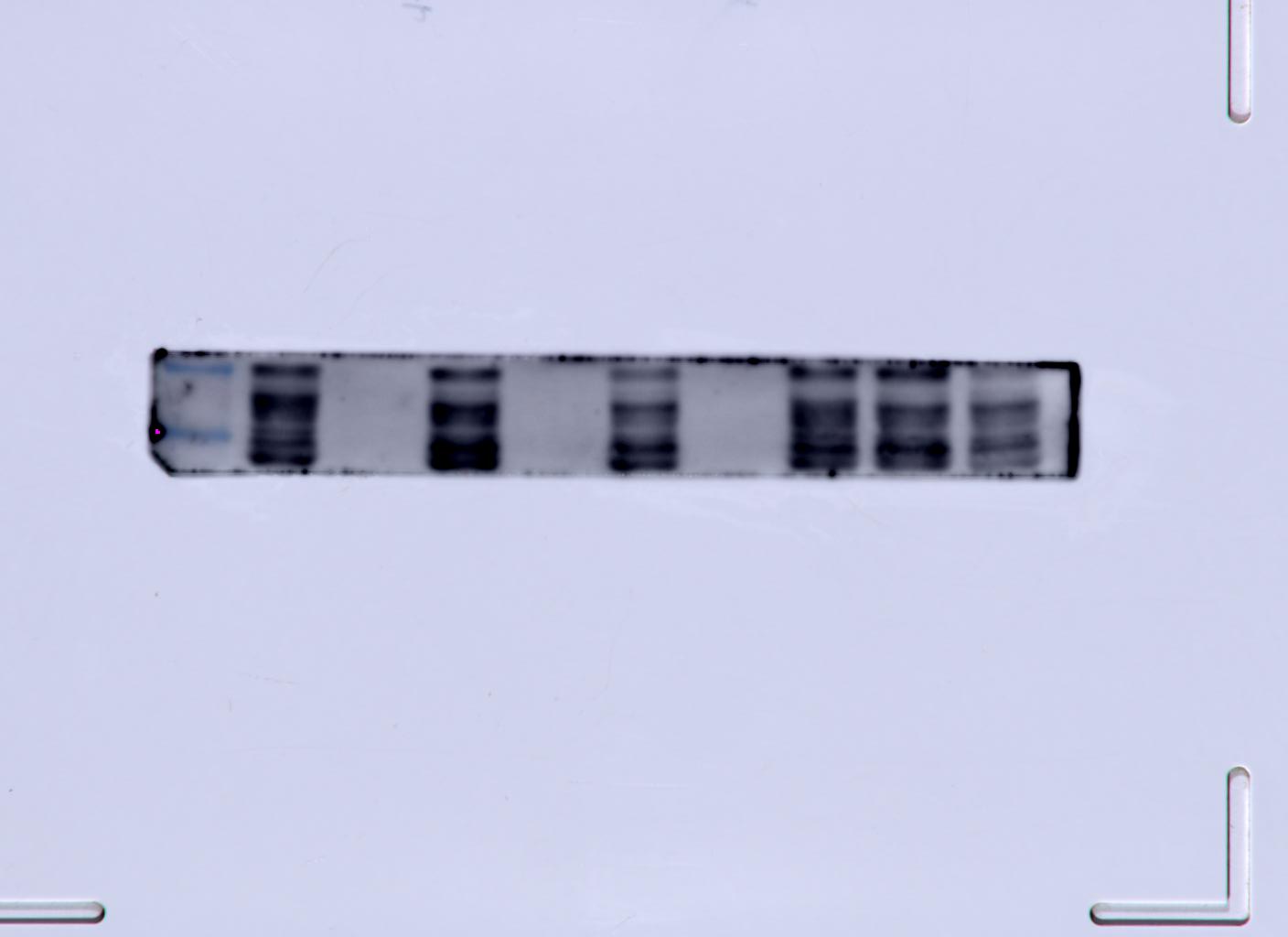


35KDa

Caput

Corpus

Cauda

CD34

C2

39KDa

48KDa


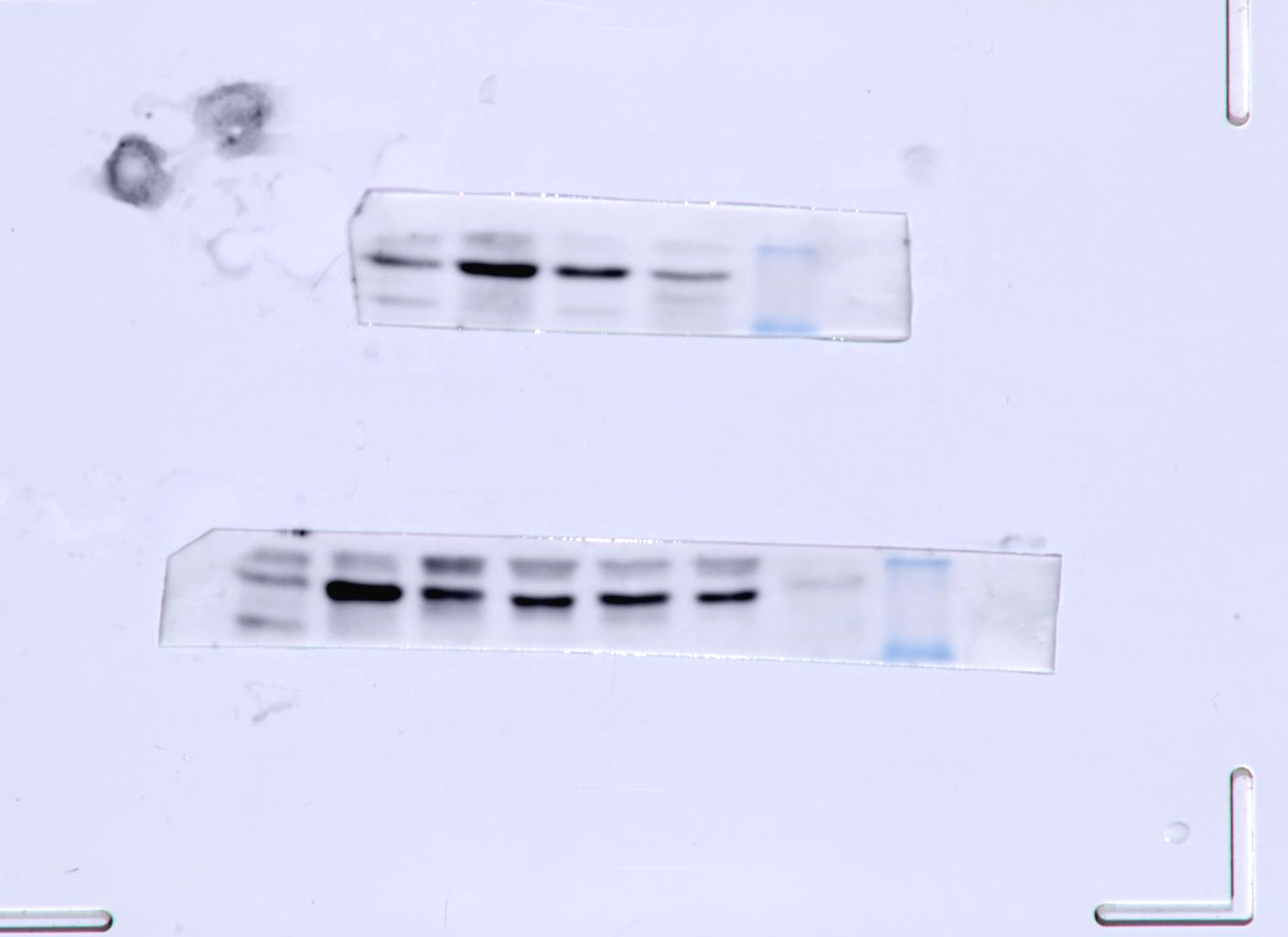


39KDa

Caput

Corpus

Cauda

CD34

C3

48KDa

35KDa

**Figure C1, C2 and C3** are the gel images of three replicate experiments of CD34 Western blot in the caput, corpus and cauda of yak epididymis.


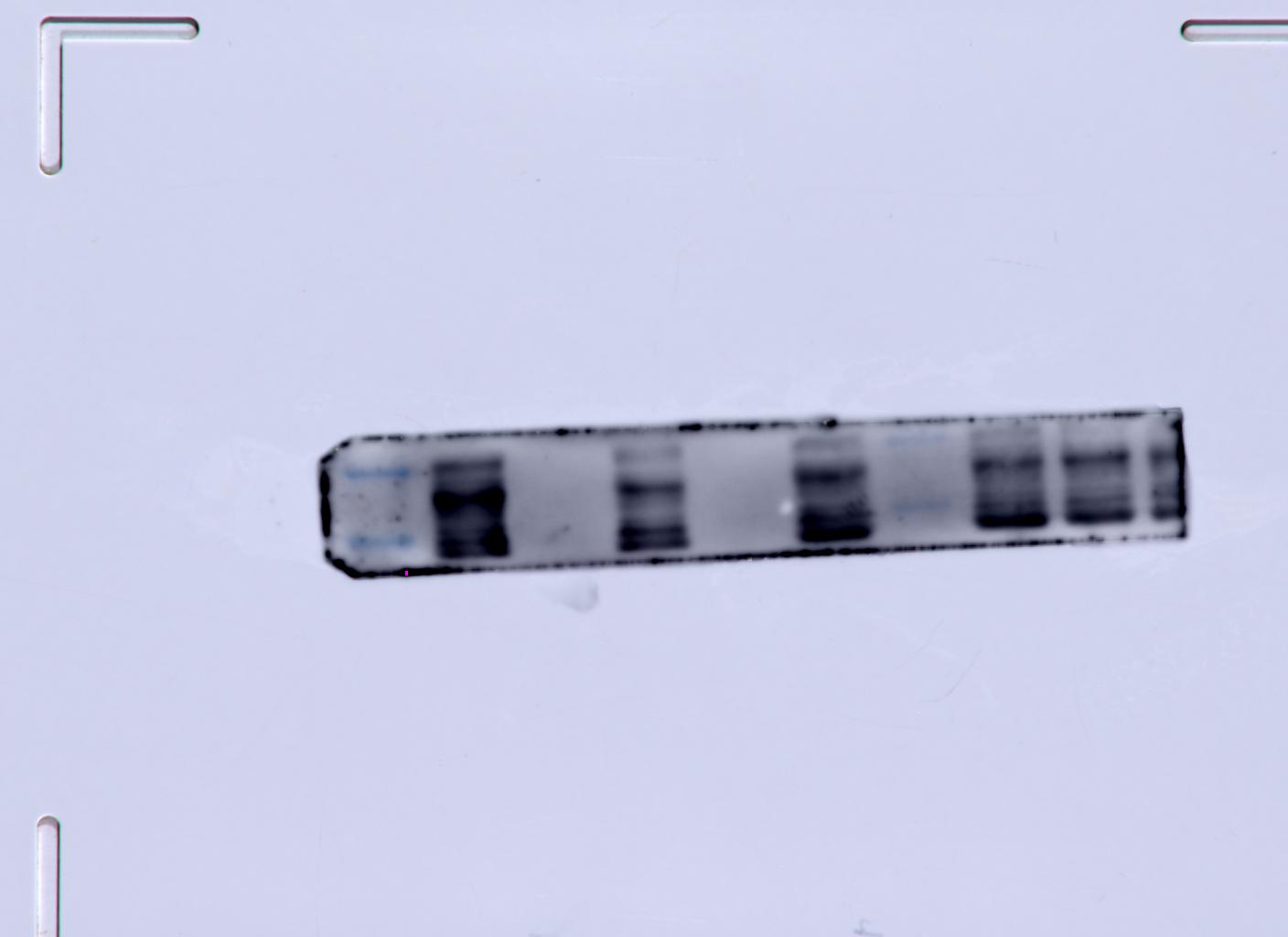


Caput

Corpus

Cauda

CD117

D1

135KDa

100KDa

105KDa


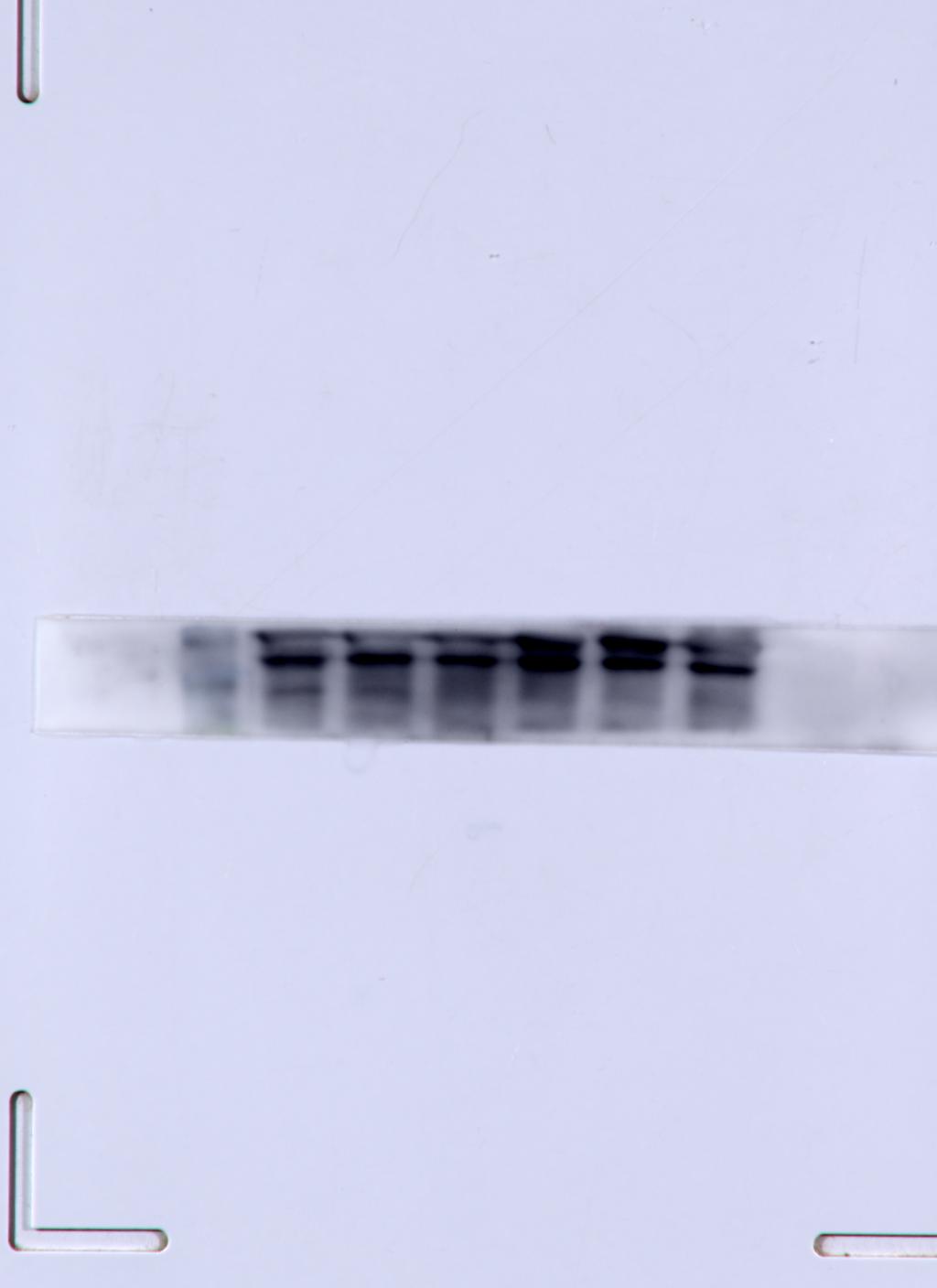


Cauda

Corpus

Caput

CD117

D2

135KDa

100KDa

100KDa


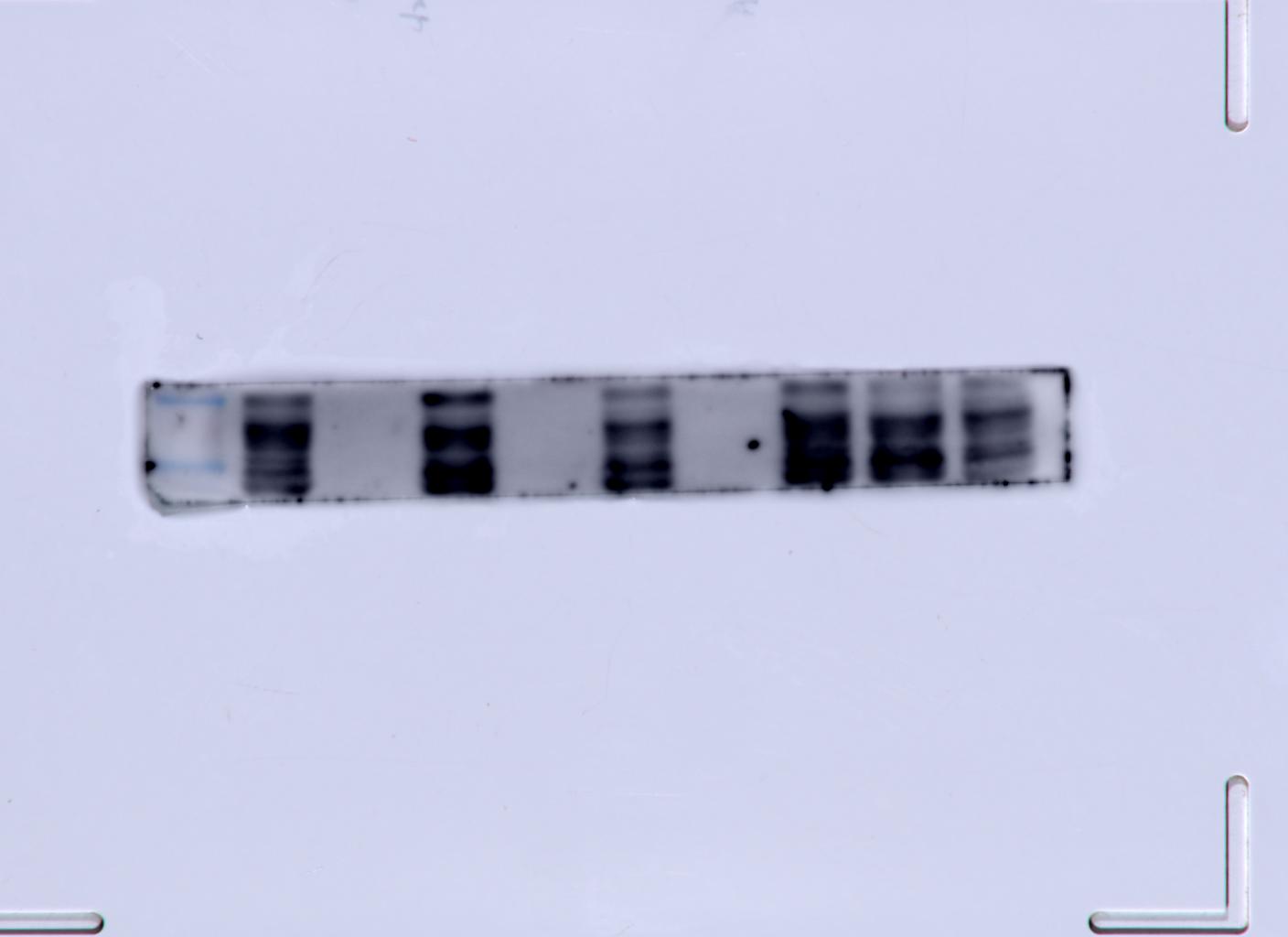


CD117

Cauda

Corpus

Caput

D3

135KDa

100KDa

105KDa

**Figure D1, D2, D3** are the gel images of three replicate experiments of CD117 Western blot in the caput, corpus and cauda of yak epididymis.
